# Superior Diagnostic Efficacy of N‐Terminal Propeptide of Type III Collagen and Golgi Protein 73 for Detection of Fibrosis in Chronic Hepatitis B Patients

**DOI:** 10.1002/mco2.70236

**Published:** 2025-06-11

**Authors:** Qianqian Chen, Ming‐Hua Zheng, Li Zhu, Fajuan Rui, Wenjing Ni, Yali Xiong, Xinyu Hu, Rahma Issa, Yixuan Zhu, Leyao Jia, Scott Barnett, Shengxia Yin, Chuanwu Zhu, Chao Wu, Mindie H. Nguyen, Jie Li

**Affiliations:** ^1^ Department of Infectious Diseases Nanjing Drum Tower Hospital Clinical College of Nanjing University of Chinese Medicine Nanjing Jiangsu China; ^2^ Department of Gastroenterology the Affiliated Huai'an Hospital of Xuzhou Medical University and Huai'an Second People's Hospital Huai'an Jiangsu China; ^3^ MAFLD Research Center Department of Hepatology the First Affiliated Hospital of Wenzhou Medical University Wenzhou Zhejiang China; ^4^ Key Laboratory of Diagnosis and Treatment for the Development of Chronic Liver Disease in Zhejiang Province Wenzhou Zhejiang China; ^5^ Department of Hepatology the Fifth People's Hospital of Suzhou Suzhou Jiangsu China; ^6^ Department of Infectious Disease Nanjing Drum Tower Hospital Affiliated Hospital of Medical School Nanjing University Nanjing Jiangsu China; ^7^ Institute of Viruses and Infectious Diseases Nanjing University Nanjing Jiangsu China; ^8^ Department of Pharmacy Ismailia Teaching Oncology Hospital Ismailia Egypt; ^9^ Department of Infectious Diseases Nanjing Drum Tower Hospital Clinical College of Nanjing Medical University Nanjing Jiangsu China; ^10^ Division of Gastroenterology and Hepatology Stanford University Medical Center Palo Alto California USA; ^11^ Department of Epidemiology and Population Health Stanford University School of Medicine Palo Alto California USA

**Keywords:** biomarker, chronic hepatitis B, Golgi protein 73, liver fibrosis, N‐terminal propeptide of type III collagen

## Abstract

Significant liver fibrosis is an indication for antiviral therapy in chronic hepatitis B (CHB). Using liver histology assessed by Scheuer system, we evaluated the diagnostic performance of PRO‐C3, GP73, and their combination for the presence of liver fibrosis, and compared them with FIB‐4, APRI, Agile 3+, FAST, and LSM in treatment‐naïve CHB patients from two centers. The study included 324 patients, of whom 167 had S2–4 (significant fibrosis) and 83 had S3–4 (advanced fibrosis). PRO‐C3 levels were higher in those with S2–4 and S3–4 compared with S0–1 and S0–2 (both *p *< 0.001), with similar findings for GP73. PRO‐C3 and GP73 were independently associated with S2–4 and S3–4 in multivariable analyses. The area under the curves (AUCs) of PRO‐C3 for S2–4 and S3–4 were 0.81 and 0.80, respectively, and exceeded those of GP73 (0.75 and 0.73). The combination of PRO‐C3 and GP73 also had significantly higher AUCs for both S2–4 (0.84 vs. 0.64) and S3–4 (0.80 vs. 0.65) as compared with FIB‐4, with similar findings for APRI, GP73, LSM, FAST, and Agile 3+ for S2–4. In conclusion, PRO‐C3 alone or in combination with GP73 is highly predictive for detecting significant liver fibrosis among CHB patients.

## Graphical abstract

Using liver histology as the gold standard, our study demonstrated that while the diagnostic performance of PRO‐C3 and GP73 is further enhanced when used in combination, PRO‐C3 alone represents a highly predictive serum biomarker for detecting significant liver fibrosis in CHB patients.

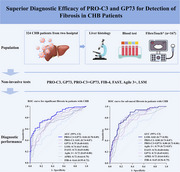

## 1 Introduction

Chronic hepatitis B (CHB) is one of the primary causes of liver fibrosis, affecting about 257 million people globally [1]. Without timely identification and intervention, CHB can progress to cirrhosis and increase risk of hepatocellular carcinoma (HCC). According to a recent estimate, the global number of CHB‐related deaths remains nearly 1 million each year [2]. Fortunately, well‐tolerated and effective oral antiviral medications are available for patients with CHB at high risk for HCC and disease progression such as those with significant fibrosis [3, 4, 5, 6].

Therefore, accurate detection of significant liver fibrosis is one of the cornerstones of CHB management. Though histological assessment remains the golden standard for diagnosing liver fibrosis, it is constrained by its invasive nature. Thus, several noninvasive tests (NITs) have been developed as potential alternatives to liver biopsy [7]. Liver stiffness measurement (LSM) using imaging‐based elastography such as magnetic resonance elastography is highly accurate, but is also limited by its low availability, while other imaging‐based techniques are device and operator dependent and susceptible to many confounders [8, 9]. Moreover, FibroScan‐aspartate aminotransferase (FAST) and Agile 3+ based on FibroScan are also restricted. Serum‐based NITs using indirect markers of liver inflammation and fibrosis such as the fibrosis‐4 index (FIB‐4) and aspartate aminotransferase (AST) to platelet (PLT) ratio index (APRI) are much more accessible, but their diagnostic accuracy for fibrosis in CHB patients is less reliable [10, 11, 12, 13]. Direct serological indicators of liver fibrosis such as hyaluronic acid, N‐terminal procollagen III‐peptide (PIIINP), and N‐terminal propeptide of type III collagen (PRO‐C3) have recently emerged as promising markers for fibrosis detection [7].

PRO‐C3, a dynamic biomarker of fibrogenesis, can be quantitatively measured using enzyme‐linked immunosorbent assay (ELISA) [14, 15]. It is generated during the synthesis of type III collagen, a major component of the extracellular matrix, and is released into the bloodstream as a byproduct of collagen turnover [16, 17]. Its presence reflects active fibrogenic activity, making PRO‐C3 a more sensitive indicator of ongoing fibrosis compared with static markers of collagen accumulation. Previous studies have indicated a close association between PRO‐C3 and liver fibrosis, particularly in patients with metabolic‐dysfunction steatotic liver disease (MASLD), [18, 19, 20] as well as in those with chronic hepatitis C (CHC) and alcoholic‐associated liver disease patients [21, 22, 23, 24].

Golgi protein 73 (GP73) is another promising tool for fibrosis detection. As a transmembrane glycoprotein localized on the Golgi apparatus, GP73 is expressed predominantly in biliary epithelial cells rather than hepatocytes [25, 26], and its serum levels closely reflect its hepatic expression [27]. This unique expression pattern makes GP73 a sensitive indicator of liver injury and fibrosis. Recent studies have reported that serum GP73 can help identify liver fibrosis and cirrhosis in patients with chronic liver diseases, including MASLD, CHC, and liver disease of mixed etiologies [27, 28, 29]. However, data on the diagnostic efficacy of PRO‐C3 and/or GP73 for liver fibrosis in CHB patients are currently limited. Our aim was to evaluate the diagnostic performance of PRO‐C3 and GP73 with other commonly used NITs.

## 2 Results

### 2.1 Patient Characteristics

**FIGURE 1:**
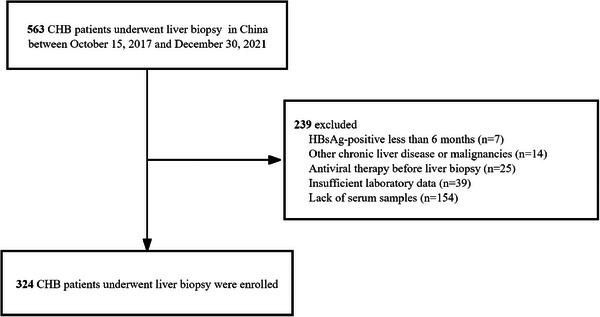
Flow diagram of the study population.

A total of 324 eligible treatment‐naïve CHB patients were included (Table 1 and Figure 1). The cohort comprised 63.6% males, with a median age of 39.0 years (interquartile range [IQR]: 32.0–47.0) and a median body mass index (BMI) of 23.64 kg/m^2^ (IQR: 21.53–25.47). Liver histologic analysis showed that 167 patients (51.5%) had significant fibrosis (S2–4), while 83 patients (25.6%) had advanced fibrosis (S3–4). LSM results were available for 167 patients, with a similar distribution of fibrosis with 90 (53.9%) having S2–4 and 50 (29.9%) having S3–4. Additional details can be found in Table S1.

**TABLE 1:** Baseline characteristics of CHB patients stratified according to liver fibrosis stages.

Variable	Overall *n* = 324	S0–1 *n* = 157	S2–4 *n* = 167	*p* Value	S0–2 *n* = 241	S3–4 *n* = 83	*p* Value
Male, *n* (%)	206 (63.6%)	100 (63.7%)	106 (63.5%)	0.967	156 (64.7%)	50 (60.2%)	0.464
Age (years)	39.0 (32.0, 47.0)	40.0 (32.5, 48.0)	38.0 (32.0, 47.0)	0.599	39.0 (33.0, 47.0)	39.0 (32.0, 48.0)	0.723
BMI (kg/m^2^)	23.64 (21.53, 25.47)	23.61(21.23, 24.87)	23.64 (21.95, 25.83)	0.097	23.64 (21.52, 25.26)	23.90 (21.55, 25.65)	0.369
WBC (10^9^/L)	5.1 (4.3, 6.1)	5.2 (4.5, 6.2)	4.9 (4.2, 5.9)	0.023	5.2 (4.4, 7.1)	4.7 (3.9, 5.6)	0.004
Hb (g/L)	146 (132, 158)	144 (131, 154)	147 (135, 161)	0.043	145 (131, 156)	150 (137, 164)	0.036
PLT (10^9^/L)	167 (136, 209)	184 (152, 219)	155 (119, 193)	<0.001	177 (146, 217)	143 (109, 177)	<0.001
TBIL (µmol/L)	12.7 (9.2, 17.2)	11.5 (8.3, 16.2)	13.6 (10.8, 17.9)	0.001	11.9 (8.7, 16.4)	14.6 (11.4, 18.7)	0.001
ALB (g/L)	42.4 (39.3, 44.9)	41.6 (38.7, 44.1)	43.0 (40.2, 46.0)	<0.001	42.0 (39.3, 44.1)	43.7 (39.2, 47.5)	0.002
ALT (U/L)	39.0 (24.9, 62.7)	36.7 (22.0, 60.2)	41.0 (26.9, 75.0)	0.033	39.0 (24.1, 60.2)	40.0 (27.0, 79.0)	0.087
AST (U/L)	26.0 (20.0, 38.0)	23.0 (19.0, 32.0)	28.0 (22.0, 43.0)	<0.001	24.0 (20.0, 33.0)	32.0 (25.0, 48.0)	<0.001
ALP (U/L)	63.2 (49.6, 79.9)	58.2 (43.3, 69.7)	69.3 (54.0, 87.0)	<0.001	61.3 (47.1, 88.1)	72.0 (57.0, 94.0)	<0.001
GGT (U/L)	28.3 (17.0, 43.2)	22.8 (16.2, 40.9)	31.4 (19.0, 51.7)	0.002	26.0 (16.2, 40.8)	36.0 (22.0, 64.0)	<0.001
HBsAg (IU/mL)	3337 (848, 14367)	3335 (680, 17114)	3345 (1122, 11675)	0.647	4391 (855, 24488)	2565 (789, 4268)	0.004
HBeAg positive, n (%)	188 (58.0%)	109 (69.4%)	79 (47.3%)	<0.001	145 (60.2%)	43 (51.8%)	0.183
HBV DNA (log IU/mL)	4 (3, 7)	3 (0, 7)	5 (3, 7)	<0.001	4 (2, 7)	4 (3, 6)	0.231
PRO‐C3 (ng/mL)	27.43 (13.21, 48.41)	16.26 (11.38, 42.01)	41.64 (22.50, 59.94)	<0.001	19.51 (12.14, 35.70)	50.82 (33.88, 66.96)	<0.001
GP73 (ng/mL)	24.82 (17.93, 38.42)	18.42 (14.22, 27.18)	30.94 (20.77, 54.98)	<0.001	20.08 (16.05 33.09)	33.31 (21.11, 67.70)	<0.001
FIB‐4	1.06 (0.68, 1.71)	0.94 (0.59, 1.39)	1.16 (0.75, 1.16)	<0.001	0.94 (0.65, 1.45)	1.43 (0.96, 2.44)	<0.001
APRI	0.4 (0.3, 0.7)	0.3 (0.2, 0.5)	0.5 (0.3, 0.8)	<0.001	0.3 (0.2, 0.5)	0.7 (0.4, 0.9)	<0.001

*p* Values, Mann–Whitney *U* test for continuous variables; Chi‐squared test for qualitative variables.

Abbreviations: CHB, chronic hepatitis B; BMI, body mass index; WBC, white blood cell; Hb, hemoglobin; PLT, platelet; TBIL, total bilirubin; ALB, albumin; ALT, alanine aminotransferase; AST, aspartate aminotransferase; ALP, alkaline phosphatase; GGT, gamma‐glutamyltransferase; HBsAg, hepatitis B surface antigen; HBeAg, hepatitis B e‐antigen; PRO‐C3, N‐terminal propeptide of type III collagen; GP73, Golgi protein 73; S, fibrosis stage; FIB‐4, fibrosis‐4 index; APRI, aspartate aminotransferase‐to‐platelet ratio index.

### 2.2 PRO‐C3 and GP73 Levels Stratified by Presence of Liver Fibrosis

PRO‐C3 levels were significantly different in both the S2–4 group and S3–4 group as compared with the group with lower staged fibrosis (Table 1), with the median value of PRO‐C3 significantly lower in S0–1 as compared with S2–4 patients (16.26 [11.38–42.01] ng/mL vs. 41.64 [22.50–59.94] ng/mL, *p* < 0.001) and in S0–2 as compared with S3–4 patients (19.51 [12.14–35.70] ng/mL vs. 50.82 [33.88–66.96] ng/mL, *p *< 0.001). As found with PRO‐C3, the median level of GP73 was significantly lower in S0–1 as compared with S2–4 patients (18.42 [14.22–27.18] ng/mL vs. 30.94 [20.77–54.98] ng/mL, *p *< 0.001) and in S0–2 as compared with S3–4 patients (20.08 [16.05–33.09] ng/mL vs. 33.31 [21.11–67.70] ng/mL, *p *< 0.001). Similar findings of higher levels of PRO‐C3 and GP73 among patients with higher fibrosis stages were also observed in the 167 patients with additional LSM data (Table S1).

### 2.3 Association Between PRO‐C3 and GP73 with Liver Fibrosis

Based on the Youden Index, cut‐off values for diagnosing S2–4 and S3–4 were 28.95 and 42.32 ng/mL for PRO‐C3 and 23.07 and 28.99 ng/mL for GP73, respectively. Subsequently, PRO‐C3 and GP73 were transformed into categorical variables based on their cut‐off values. Following adjustment for sex, age, BMI, liver enzymes, and HBV DNA levels, multivariate logistic regression analysis revealed that PRO‐C3 and GP73 were significant and strong independent factors associated with S2–4 (adjusted odds ratio [aOR] 4.67 95% confidence interval [CI]: 2.74–7.96 and aOR 2.82 95% CI: 1.64–4.85, respectively, both *p *< 0.001) as well as S3–4 (aOR 8.37 95% CI: 4.17–16.78 and 3.91 95% CI: 2.04–7.49, respectively, both *p *< 0.001) (Table 2).

**TABLE 2:** Univariate and multivariate logistic regression analysis of CHB patients with significant fibrosis (S2–4) and advanced fibrosis (S3–4).

Variable	Significant fibrosis (S2–4)				Advanced fibrosis (S3–4)			
	Univariate analysis		Multivariate analysis		Univariate analysis		Multivariate analysis	
	OR (95% CI)	*p* Value	aOR (95% CI)	*p* Value	OR (95% CI)	*p* Value	aOR (95% CI)	*p* Value
Male (%)	1.01 (0.64–1.59)	0.97			1.21 (0.73–2.02)	0.46		
Age (years)	1.00 (0.97–1.02)	0.64			1.01 (0.98–1.03)	0.52		
BMI (kg/m^2^)	1.06 (0.99–1.14)	0.10			1.05 (0.97–1.13)	0.24		
ALT (U/L)	1.00 (1.00‐1.00)	0.57			1.00 (1.00‐1.00)	0.65		
AST (U/L)	1.00 (1.00–1.01)	0.18			1.00 (1.00–1.01)	0.36		
ALP(U/L)	1.03 (1.02–1.04)	<0.001	1.02 (1.01–1.03)	<0.01	1.02 (1.01–1.03)	<0.001	1.02 (1.01–1.03)	0.01
GGT(U/L)	1.01 (1.00–1.02)	<0.01			1.02 (1.01–1.02)	<0.001		
HBeAg positive (%)	2.53 (1.60–3.99)	<0.001	1.96 (1.12–3.43)	0.02	1.41 (0.85–2.32)	0.18		
HBV DNA (log IU/mL)	1.19 (1.93–1.30)	<0.001			1.07 (0.97–1.18)	0.16	0.84 (0.73–0.96)	0.01
PRO‐C3	6.85 (4.20–11.15)	<0.001^a^	4.67 (2.74–7.96)	<0.001^a^	9.58 (5.44–16.87)	<0.001^b^	8.37 (4.17–16.78)	<0.001^b^
GP73	4.73 (2.96–7.56)	<0.001^c^	2.82 (1.64–4.85)	<0.001^c^	5.99 (3.48–10.30)	<0.001^d^	3.91 (2.04–7.49)	<0.001^d^

Abbreviations: CHB, chronic hepatitis B; S, fibrosis stage; OR: odds ratio; CI, confidence interval; aOR: adjusted odds ratio; BMI, body mass index; ALT, alanine aminotransferase; AST, aspartate aminotransferase; ALP, alkaline phosphatase; GGT, gamma‐glutamyltransferase; HBeAg, hepatitis B e‐antigen; PRO‐C3, N‐terminal propeptide of type III collagen; GP73, Golgi protein 73.

^a^ PRO‐C3 >28.95 ng/mL.

^b^ PRO‐C3 >42.32 ng/mL.

^c^ GP73 >23.07 ng/mL.

^d^ GP73 >28.99 ng/mL.

### 2.4 Diagnostic Efficacy of PRO‐C3 and GP73 for Liver Fibrosis

**FIGURE 2:**
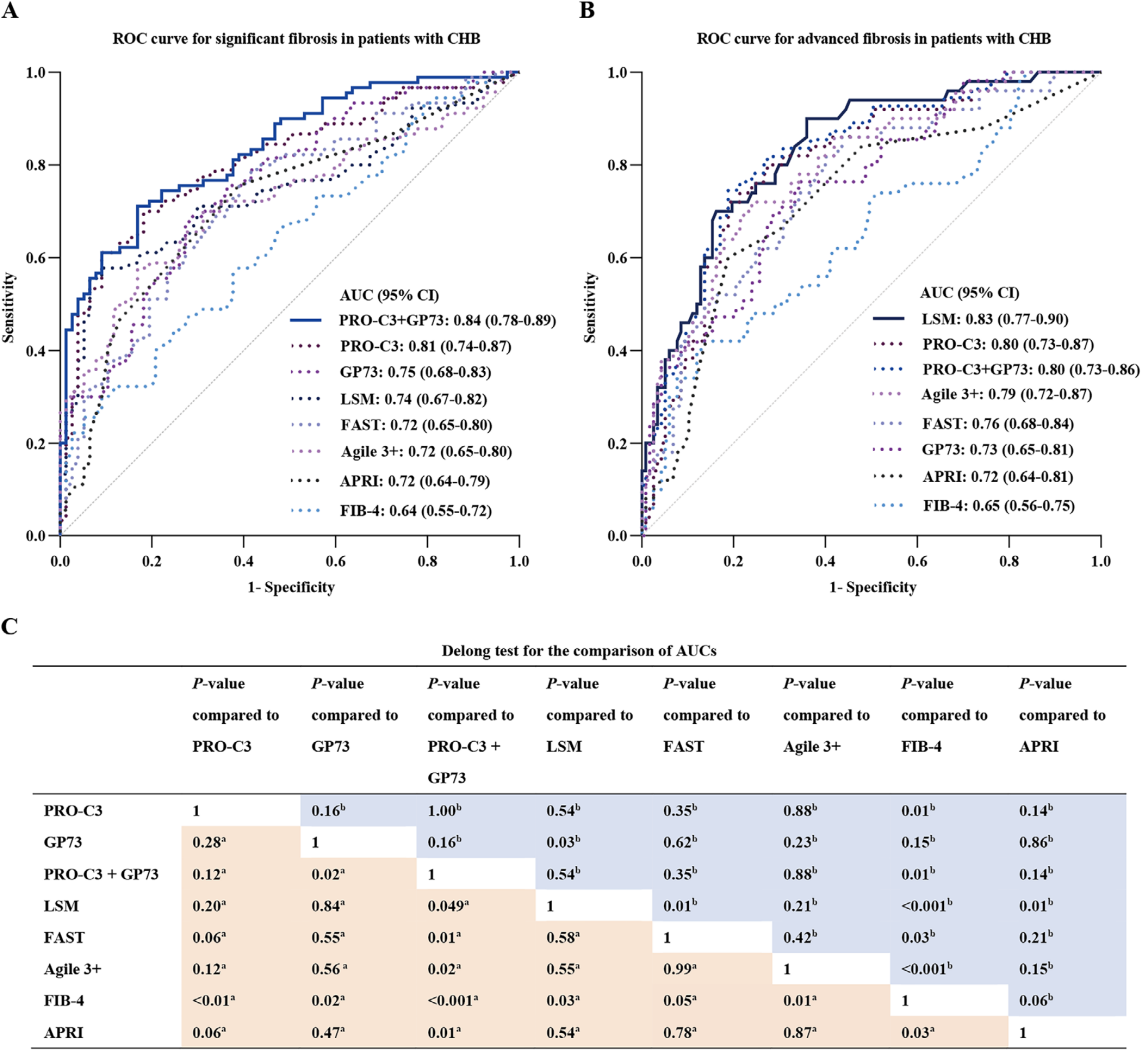
Comparison of diagnostic efficacy for identifying fibrosis in CHB patients. ROC curves for identifying significant fibrosis (S2–4) (A) and advanced fibrosis (S3–4) (B). (C) Delong test for the differences between AUCs of the different NITs. *Note*: a, significant fibrosis; b, advanced fibrosis. Logistic regression was performed to calculate predicted values of PRO‐C3+GP73.

In this study, three diagnostic models were built separately based on PRO‐C3 alone, GP73 alone, and a combination of PRO‐C3 and GP73 (Figure 2 and Table S2). The areas under the curves (AUCs) for diagnosing S2–4 and S3–4 were 0.81 (0.74–0.87) and 0.80 (0.73–0.87) for PRO‐C3 and 0.75 (0.68–0.83) and 0.73 (0.65–0.81) for GP73, respectively. The combination of PRO‐C3 and GP73 demonstrated the highest diagnostic efficacy with AUC of 0.84 (0.78–0.89) for S2–4, which was significantly higher than those of FIB‐4 (0.64 [0.55–0.72], *p *< 0.001), APRI (0.72 [0.64–0.79], *p *= 0.01), Agile 3+ (0.72 [0.65–0.80], *p *= 0.02), FAST (0.72 [0.65–0.80], *p *= 0.01), LSM (0.74 [0.67–0.82], *p *= 0.049) and GP73 (*p *= 0.02) but not of PRO‐C3 alone (Figure 2A,C). For S3–4, there was no statistically significant difference of the AUCs between PRO‐C3 and LSM or between the PRO‐C3+GP73 prediction model and other models, except for FIB‐4 having a significantly lower AUC (Figure 2B,C).

### 2.5 Decision Curve Analysis and Calibration Plot of Diagnostic Models

**FIGURE 3:**
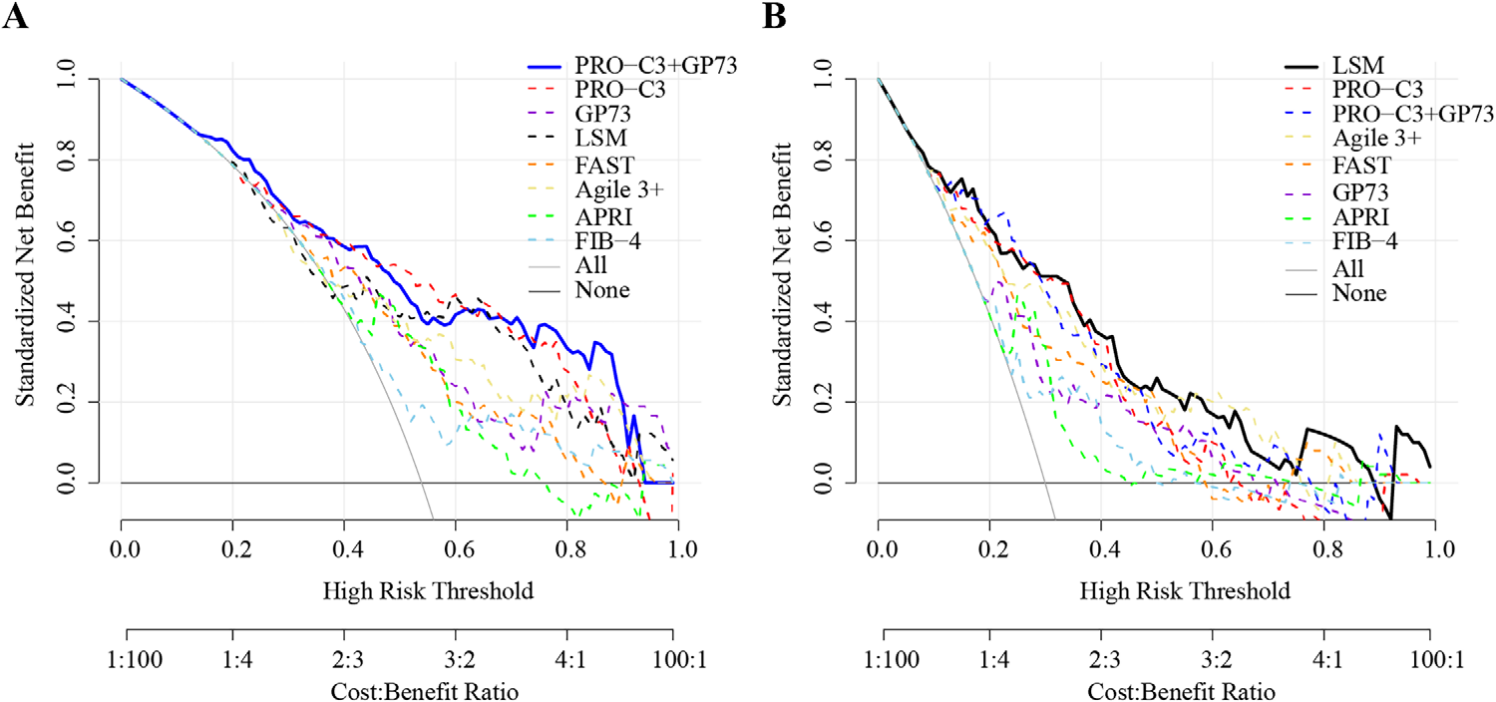
DCA for identifying fibrosis in CHB patients. Decision curves of different NITs for identifying significant fibrosis (S2–4) (A) and advanced fibrosis (S3–4) (B). *Note*: Logistic regression was performed to calculate predicted values of PRO‐C3 + GP73.

**FIGURE 4:**
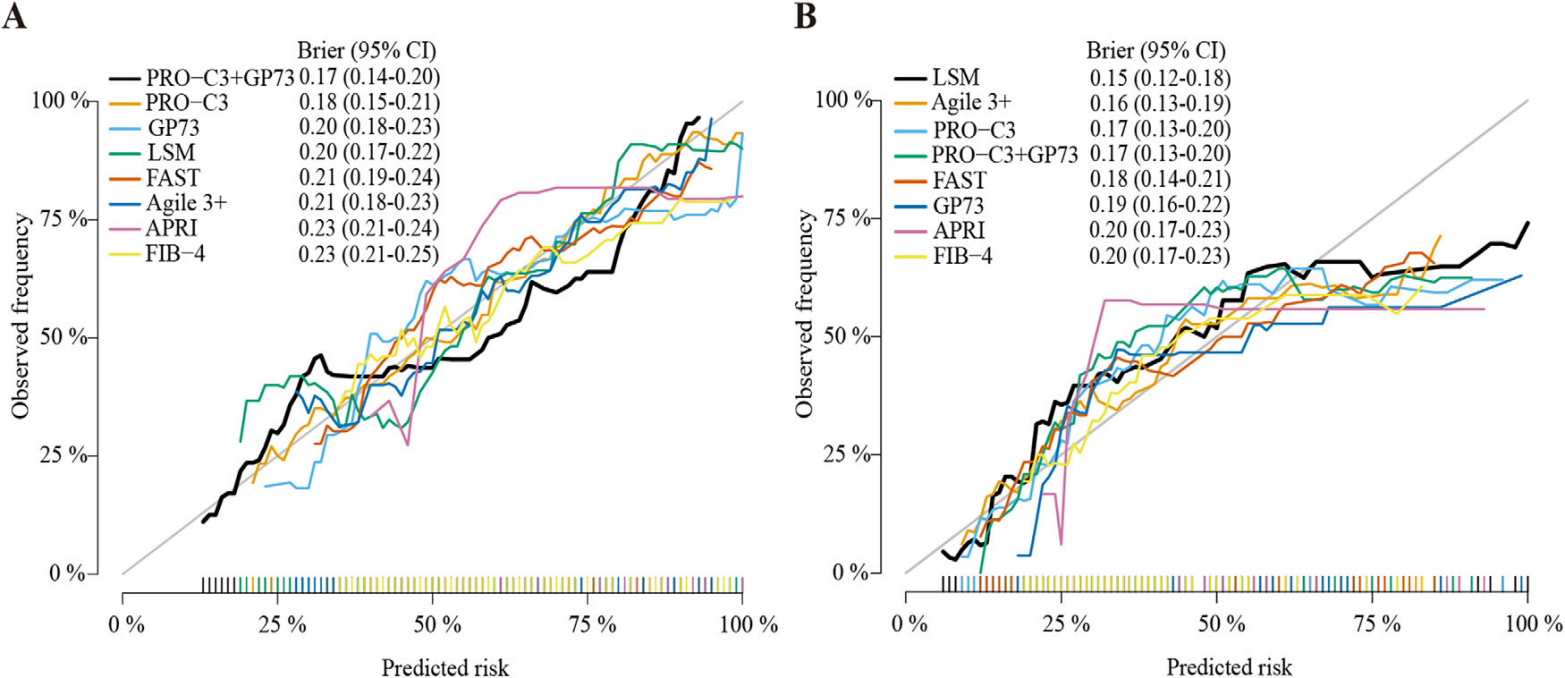
Calibrations for identifying fibrosis in CHB patients. Calibrations of different NITs for identifying significant fibrosis (S2–4) (A) and advanced fibrosis (S3–4) (B). Note: Logistic regression was performed to calculate predicted values of PRO‐C3+GP73.

To further elucidate their diagnostic value and clinical net benefit, decision curve analysis (DCA) was applied for these diagnostic tools (Figure 3). The PRO‐C3 + GP73 combination model demonstrated a superior diagnostic curve for identifying S2–4 compared with other models and the default strategies of performing liver biopsy in all patients, or none, indicated superior net benefit across most threshold probabilities (Figure 3A). The decision curve for the PRO‐C3 + GP73 combination model for S3–4 closely paralleled the PRO‐C3 curve, but was lower than the LSM curve (Figure 3B). Calibration curves showed that the Brier scores for the PRO‐C3 and GP73 were 0.18 (0.15–0.21) and 0.20 (0.18–0.23) in S2–4 group, respectively. The PRO‐C3 + GP73 combination showed high accuracy with lower Brier score (0.17 [0.14–0.20]) compared with those of other diagnostic tools (LSM: 0.20 [0.17–0.22]; FAST: 0.21 [0.19–0.24]; Agile 3+: 0.21 [0.18–0.23]; APRI: 0.23 [0.21–0.24]; FIB‐4: 0.23 [0.21–0.25]) (Figure 4A). In the calibration curve of S3–4, the Brier scores for the PRO‐C3 and PRO‐C3+GP73 combination models were both 0.17 (0.13–0.20) and had high accuracy compared with 0.20 (0.17–0.23) for FIB‐4 and APRI, and 0.18 (0.14–0.21) for FAST (Figure 4B).

### 2.6 Subgroup Analysis

**FIGURE 5:**
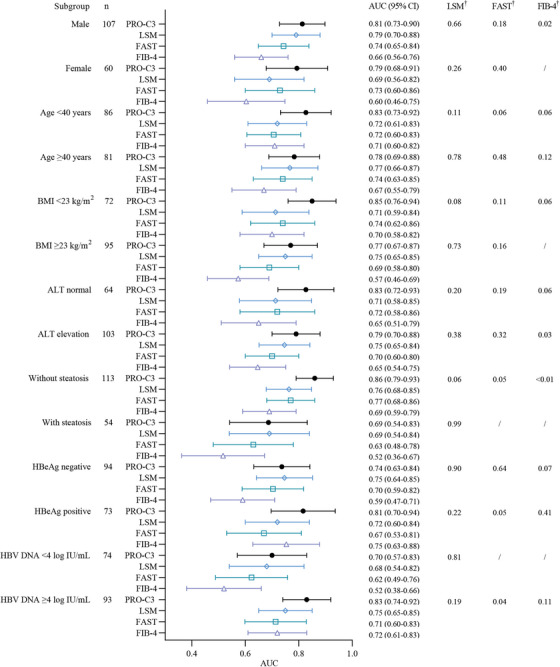
Forest plot for the AUCs of different NITs for identifying significant fibrosis (S2–4) under different subgroups by gender, age (years), BMI (kg/m^2^), ALT (U/L), steatosis, HBeAg and HBV DNA (log IU/mL). ALT normal, ALT ≤35 U/L in the male, ALT ≤25 U/L in the female; ALT elevation, ALT >35 U/L in the male, ALT >25 U/L in the female. †:P‐value was calculated by Delong test for pairwise comparison of the AUCs in PRO‐C3 and other NITs (LSM, FAST, and FIB‐4). “/” indicates no comparison was performed due to no statistical difference for AUC in one subgroup.

**FIGURE 6:**
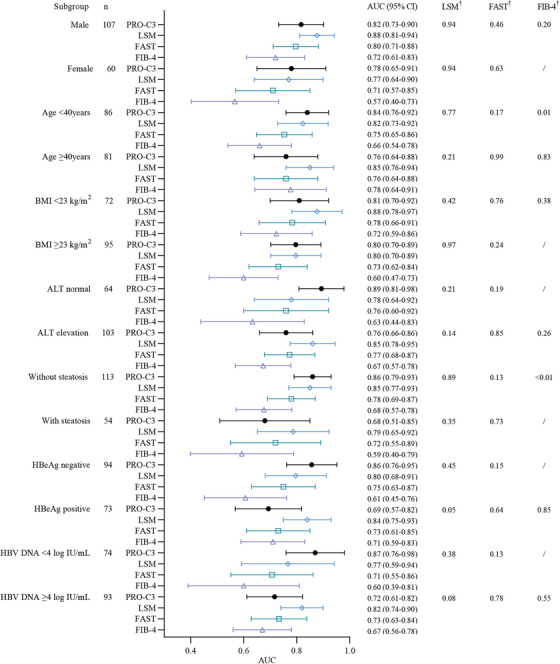
Forest plot for the AUCs of different NITs for identifying advanced fibrosis (S3–4) under different subgroups by gender, age (years), BMI (kg/m^2^), ALT (U/L), steatosis, HBeAg and HBV DNA (log IU/mL). ALT normal, ALT ≤35 U/L in the male, ALT ≤25 U/L in the female; ALT elevation, ALT >35 U/L in the male, ALT >25 U/L in the female. †:P‐value was calculated by Delong test for pairwise comparison of the AUCs in PRO‐C3 and other NITs (LSM, FAST, and FIB‐4). “/” indicates no comparison was performed due to no statistical difference for AUC in one subgroup.

Subgroup analyses were performed based on sex, age, BMI, alanine aminotransferase (ALT) level, presence of hepatic steatosis, hepatitis B e‐antigen (HBeAg) status, and HBV DNA levels. with consistent findings as in main analyses across most subgroups in both multivariable regression analyses (Tables S3 and S4) and comparative analyses of AUCs (Figures 5 and 6 and Tables S5, S6, and S7). The subgroup analyses showed that the diagnostic performance of PRO‐C3 combined with GP73 was superior to FIB‐4 and APRI, but maintained similar competence with LSM, Agile 3+, and FAST for detection of S2–4 among most subgroups.

## 3 Discussion

Using liver histology as the gold standard, our two centers, cross‐sectional study revealed a significant and strong association between high PRO‐C3 and GP73 levels in CHB patients with significant liver fibrosis, a finding that was also consistent in most demographic and clinical subgroups. Though the diagnostic performance of PRO‐C3 and GP73 is further enhanced when used in combination, PRO‐C3 alone had superior performance to FIB‐4 (AUC = 0.81 [0.74–0.87] vs. 0.64 [0.55–0.72], *p *< 0.01) and similar diagnostic performance as LSM (AUC = 0.74 [0.67–0.82], *p *= 0.20), FAST (AUC = 0.72 [0.65‐0.80], *p *= 0.06), Agile 3+ (AUC = 0.72 [0.65–0.80], *p *= 0.12), and APRI (AUC = 0.72 [0.64–0.79], *p *= 0.06). Furthermore, while suboptimal performance of LSM and FIB‐4 have been reported for various subgroups by age, BMI, and diabetes [30, 31], our study found that the performance of PRO‐C3 was consistent across different subgroups.

Our current study built on prior literature reporting the association of PRO‐C3 with the presence and progression of liver fibrosis in patients with MASLD [20, 32, 33]. Prior studies have also reported the link between PRO‐C3 and liver fibrosis as well as fibrosis progression in patients with CHC [19, 21, 22]. As previous studies in other populations, we found a strong and significant association between PRO‐C3 and the presence of significant as well as advanced fibrosis in patients with CHB. This finding was in contrast with a study which found no significant association between elevated PRO‐C3 level and liver fibrosis in their cohort of 96 treatment‐naïve CHB patients, which our surmise was due to its small study sample size [34]. Our study also adds to the current knowledge in the association between elevated serum GP73 levels and liver fibrosis in patients with CHC [29] and expands it to the CHB population. These findings are in line with prior studies reporting association between GP73 levels and other liver‐specific biomarkers such as hyaluronic acid, laminin, and type III collagen [35], and that the elevation of GP73 in hepatocytes is reversible during the regression of liver fibrosis [36].

Currently, FIB‐4 is recommended as one of the first line NITs for the evaluation of liver fibrosis due to its low cost and widespread availability [37]. FIB‐4 can be easily calculated using readily available clinical markers (age, AST, ALT, and PLT), and has reasonable diagnostic performance for liver fibrosis overall. However, it has several major limitations. First, FIB‐4 is more useful to rule out fibrosis at low cutoff value due to its high negative predictive value at that level, while its usage for ruling in significant and advanced fibrosis is limited. Second, FIB‐4 performs poorly in populations that have metabolic disease such as those with obesity and/or diabetes [30]. Third, FIB‐4 can be affected by presence of liver inflammation with AST and ALT being part of formula [31]. FIB‐4 is also not reliable in adolescents and old populations [37]. On the other hand, our study found that, in addition to having superior diagnostic performance overall as compared with FIB‐4 (AUC = 0.81 [0.74–0.87] vs. 0.64 [0.55–0.72], *p *< 0.01), PRO‐C3 had consistent performance across most demographic and clinical subgroups to include age, sex, BMI, HBeAg status, ALT, and HBV DNA levels. The addition of GP73 also further increased the performance of PRO‐C3 for the diagnosis of liver fibrosis, though the added cost of an additional biomarker test needs to be evaluated. Moreover, the combination of PRO‐C3 and GP73 outperformed APRI (AUC = 0.72 [0.64–0.79], *p *= 0.01) for detecting significant fibrosis.

Notably, the updated 2024 World Health Organization (WHO) guidelines for CHB management also recommend a NIT as the first‐line test of the evaluation of liver fibrosis in CHB patients [6]. Our findings align with the guideline's emphasis on the importance of NITs for monitoring liver fibrosis and highlight the potential of novel biomarkers, such as PRO‐C3 and GP73, to enhance risk stratification and inform treatment decisions in CHB management.

In our analysis of patients who also underwent LSM testing, the diagnostic performance of PRO‐C3 was found to be comparable to those with LSM, FAST, and Agile 3+, which were the combination of simple clinical parameters, LSM, and controlled attenuation parameter. Although Agile 3+ and FAST have been proven to be the accurate NITs of liver fibrosis for MASLD patients [38, 39, 40], PRO‐C3, a blood‐based ELISA test, may be more convenient for patients as it can be done with other routine clinical labs. Additionally, availability of LSM may be limited in primary care community settings as well as in resource limited regions. A blood‐based test can help decrease disparities in care gaps among the different practice settings.

Our study has several strengths. This cross‐sectional study used a relatively large sample size to evaluate the performance of two novel NITs (PRO‐C3 and GP73) for the detection of liver fibrosis for patients with CHB. All patients underwent liver biopsy and blood collection for PRO‐C3 and GP73 determination on the same day, limiting biases from fluctuating biomarker levels. Liver histologic analysis was also independently performed by two experienced pathologists who were blinded to patient clinical characteristics to minimize misclassification biases. However, there are several limitations in this study. First, all patients in this study are of Asian descent and the generalizability of our results to other racial ethnic groups requires additional validation. Second, although all patients in our study underwent liver biopsy and blood collection for PRO‐C3 and GP73 determination, only 167 CHB patients underwent LSM measurements. Though we also found higher PRO‐C3 and GP73 levels among patients with higher fibrosis stages in this smaller cohort of patients with LSM data, we were not able to do additional analyses for this subgroup and future studies with larger sample size of patients with available LSM data are needed. Additionally, also due to insufficient sample size, we did not further explore the efficacy of PRO‐C3 in diagnosing earlier or intermediate fibrosis stages (e.g., S1). Given the clinical importance of early detection and intervention in CHB management, future studies are needed to investigate the performance of PRO‐C3 and GP73 in distinguishing mild fibrosis from more advanced stages, as this could further refine their clinical utility. Third, while we followed a standardized procedure of sample collection across study centers, other potential confounding factors, such as stress, diurnal variation, or medication use, might influence PRO‐C3 and GP73 levels. Fourth, the lack of consensus for pathological readings in this study may introduce variability in fibrosis stages. However, we assessed interobserver variability at each study center and found a good agreement. Future studies with consensus histologic reading will improve the quality of liver biopsy reading. Finally, our study did not evaluate correlation between PRO‐C3 and GP73 with liver‐related adverse clinical outcomes, such as decompensated cirrhosis, and HCC, which should be examined in future studies.

In conclusion, PRO‐C3 and GP73 levels are strongly and significantly associated with the presence of significant and advanced liver fibrosis among patients with CHB. The diagnostic performance of PRO‐C3 for the diagnosis of fibrosis is superior to FIB‐4 and similar to LSM, FAST, Agile 3+, and APRI. As such, PRO‐C3 is a promising convenient blood‐based NIT for fibrosis in CHB patients. The diagnostic performance of PRO‐C3 is also consistent across CHB subgroups by age, sex, BMI, presence of hepatic steatosis and markers of hepatic inflammation or viral activities such as ALT or HBV DNA levels.

## 4 Methods

### 4.1 Study Design and Population

This cross‐sectional study was conducted at two medical centers in China (Nanjing Drum Tower Hospital and the Fifth People's Hospital of Suzhou) between October 15, 2017 and December 30, 2021. The definition of CHB was a positive hepatitis B surface antigen (HBsAg) for at least 6 months. Treatment‐naïve CHB patients aged 18 years or older who underwent liver biopsy were consecutively enrolled. Exclusion criteria included individuals with other forms of viral hepatitis, drug‐induced hepatitis, autoimmune hepatitis, or HCC.

### 4.2 Liver Fibrosis Assessment and Subgroup Definition

Liver histology was evaluated by two experienced pathologists from each center who were blinded to the clinical data. Each pathologist independently assessed all specimens, with any discrepancies resolved through discussion to reach a consensus. To assess interobserver variability, the agreement between pathologists was evaluated (details were provided in Materials and Methods, Supporting Information). Liver fibrosis was categorized by four stages (S0–S4) according to Scheuer system [41]. Nonsignificant fibrosis and nonadvanced fibrosis were defined as S0–1 and S0–2, respectively. Significant and advanced fibrosis were defined as S2–4 and S3–4, respectively. CHB patients were categorized into two groups: S0–1 vs. S2–4, and S0–2 vs. S3–4.

### 4.3 Clinical Parameters

The clinical variables evaluated encompassed age, sex, BMI, as well as measurements of white blood cell, hemoglobin, PLT, ALT, AST, alkaline phosphatase, gamma‐glutamyltransferase, albumin, total bilirubin, HBV DNA, HBeAg, and HBsAg. NITs for liver fibrosis assessment, including PRO‐C3, GP73, LSM, FAST, Agile 3+, and FIB‐4, were also recorded. PRO‐C3 (Nordic Bioscience, Herlev, Denmark) and GP73 (Hotgen Biotech Inc., Beijing, China) levels were determined by ELISA, and LSM was performed using FibroTouch (Wuxi Hisky Medical Technology Co., Ltd., Wuxi, China). FAST, Agile 3+, APRI, and FIB‐4 were calculated using the reported formulas [11, 38, 39]. Detailed NIT formulas were provided in Materials and Methods (Supporting Information). Ethylenediaminetetraacetic acid plasma was collected from fasting patients at the time of liver biopsy and stored at −80°C for subsequent analysis. Laboratory tests and LSM were performed according to standardized protocols at each participating center. To minimize potential variability in biomarker levels, all participants were instructed to fast for at least 8 h and refrain from strenuous exercise for 24 h prior to blood collection. Blood samples were collected in the morning under standardized conditions to mitigate the effects of diurnal variation and acute stress.

### 4.4 Sample Size Calculation

According to a systematic review and meta‐analysis [15], the AUC of PRO‐C3 for detecting significant and advanced fibrosis in patients with viral hepatitis or MASLD was approximately 0.80. Another meta‐analysis reported that the AUCs of LSM for diagnosing significant fibrosis and advanced fibrosis were 0.88 and 0.91, respectively [9]. The sample size was calculated based on a two‐sided *α* of 0.05 and a *β* of 0.2, as well as a negative: positive group sample size ratio of 1, indicating that a minimum of 100 samples was required to achieve adequate statistical power. To account for potential dropouts or incomplete data and to allow for relevant subgroup analyses, 167 patients were ultimately enrolled to ensure robust and reliable results.

### 4.5 Statistical Analyses

Statistical analyses were executed using SPSS version 25.0 (SPSS, Armonk, NY: IBM Corp) and MedCalc 19.8 (MedCalc, Ostend, Belgium: MedCalc Software Ltd). Graphical representations were created with GraphPad Prism 9 (GraphPad Prism, Boston, MA: GraphPad Software, LLC) and R 4.3.3 (http://www.R‐project.org). Continuous variables were reported as mean ± standard deviation or median (IQR: Q1, Q3), respectively. Categorical variables were expressed as frequencies and percentages. Comparative analysis of participant characteristics was conducted using one‐way ANOVA or the Mann–Whitney *U* test for continuous variables and the Chi‐square test for categorical variables, as appropriate. The cutoff values of PRO‐C3 and GP73 for diagnosing S2–4 and S3–4 were determined using the Youden Index, which were subsequently transformed into categorical variables based on their cut‐off values for logistic regression analysis. Univariate analyses identified variables statistically significantly different between patients with and without S2–4 or S3–4. Subsequent multivariate analyses employed a stepwise forward method, with entry and stay *p* values set at 0.1 and 0.05, respectively.

To evaluate the diagnostic accuracy of fibrosis markers, the AUCs were calculated. These AUCs specifically assessed the diagnostic performance of these tools for identifying S2–4 and S3–4. The predictive values of PRO‐C3 and GP73 combination models for S2–4 or S3–4 were generated by establishing a logistic regression equation, using the presence of S2–4 or S3–4 fibrosis as the binary outcome variable and incorporating both PRO‐C3 and GP73 as covariates. Comparisons between different AUCs were conducted using the method proposed by DeLong et al. [42].

DCA was conducted to evaluate clinical efficacy utility of different NITs by quantifying the net benefits under different threshold probabilities [43, 44]. The predictive models evaluated by DCA were compared with the default strategies for liver biopsy in all patients, or none. In our study, net benefit refers to a composite of the benefit obtained by performing liver biopsy‐proven S2–4/S3–4 in patients defined as S2–4/S3–4 according to NITs (true positive) and risk caused by liver biopsy in those without S2–4/S3–4 but who were defined as S2–4/S3–4 according to NITs (false positive). The threshold probability signifies a hypothetical risk level at which the anticipated benefits of undergoing a liver biopsy are equivalent to the anticipated benefits of abstaining from the procedure. The net benefit threshold is 0–1, and the greater clinical benefit is recognized with the higher net benefit score. Moreover, calibration of prediction models was employed to evaluate the diagnostic accuracy of the NITs [45]. Calibration plot is a visual tool to assess the consistency between predictions and observations in different percentiles of the predicted values. In the calibration plot, the horizontal and vertical coordinates represent the predicted and actual probability, respectively. The diagonal line in the graph refers to the concordance between the predicted and actual incidence probability under ideal conditions. A calibration Brier score of 0 suggests perfect accuracy while a score of 1 suggests perfect inaccuracy. A two‐tailed *p* value of less than 0.05 was deemed to reflect statistical significance.

## Author Contributions

Study concept and design: J. L., M. H. N., and C. W. Data acquisition and technical support: Q. C., M. H. Z., L. Z., F. R., W. N., Y. X., X. H., R. I., Y. Z., L. J., S. B., S. Y., and C. Z. Manuscript drafting: Q. C., X. H., and W. N. Critical revision of the manuscript: J. L. and M. H. N. Data access and verification: J. L. and M. H. N. All authors reviewed and approved the final manuscript.

## Ethics Statement

The study protocol was reviewed and approved by the Ethics Committees of Nanjing Drum Tower Hospital (No: 2008022) and the Fifth People's Hospital of Suzhou (No: szst‐p‐2010‐2, September 2010). Written informed consent was obtained from all participants prior to their inclusion in the study.

## Conflicts of Interest

The authors declare no conflicts of interest.

## Supporting information



Supporting Information

## Data Availability

Data are not available due to privacy issues.
