# Supplementary material for: Superior Diagnostic Efficacy of N‐Terminal Propeptide of Type III Collagen and Golgi Protein 73 for Detection of Fibrosis in Chronic Hepatitis B Patients
Source: MedComm (2020). 2025 Jun 11;6(6):e70236. doi: 10.1002/mco2.70236 (PMC12152426; doi:10.1002/mco2.70236)
Supplement: Supplementary file 1 — Supporting Information [file MCO2-6-e70236-s001.docx]

**SUPPLEMENTARY MATERIALS AND METHODS**

**Table of Content**

**Supplementary methods**

**Reference**

**Supplementary Table 1.** Baseline characteristics of CHB patients with available LSM stratified according to liver fibrosis stages

**Supplementary Table 2.** Diagnostic accuracy of different NITs for significant fibrosis (S2-4) and advanced fibrosis (S3-4) in CHB patients

**Supplementary Table 3.** Multivariate logistic regression analysis of PRO-C3 of significant fibrosis (S2-4) and advanced fibrosis (S3-4) under different subgroups by gender, age (years), BMI (kg/m^2^), ALT (U/L), steatosis, HBeAg and HBV DNA (log IU/mL)

**Supplementary Table 4.** Multivariate logistic regression analysis of GP73 of significant fibrosis (S2-4) and advanced fibrosis (S3-4) under different subgroups by gender, age (years), BMI (kg/m^2^), ALT (U/L), steatosis, HBeAg and HBV DNA (log IU/mL)

**Supplementary Table 5.** Diagnostic accuracy of different NITs for significant fibrosis under different subgroups by gender, age (years), BMI (kg/m^2^), ALT (U/L), steatosis, HBeAg and HBV DNA (log IU/mL)

**Supplementary Table 6.** Diagnostic accuracy of different NITs for advanced fibrosis under different subgroups by gender, age (years), BMI (kg/m^2^), ALT (U/L), steatosis, HBeAg and HBV DNA (log IU/mL)

**Supplementary Table 7.** Delong test for the differences between AUCs of the different NITs under different subgroups by gender, age (years), BMI (kg/m^2^), ALT (U/L), steatosis, HBeAg and HBV DNA (log IU/mL)

**Supplementary methods**

**Interobserver variability for liver histology reading**

To enhance the diagnostic reliability for significant and advanced fibrosis, we selected cases from the two cohorts included in this study and assessed interobserver variability between the two board-certified pathologists (G.Z., Z.F.) who conducted the pathological evaluations, following the methodology outlined in our prior research.^1^ Interobserver variability was evaluated using kappa statistics, with the level of agreement categorized as follows: 0%–20% (poor), 21%–40% (fair), 41%–60% (moderate), 61%–80% (good), and 81%–100% (excellent).^2^ The kappa statistic, computed as (Po - Pe) / (1 - Pe), where Po represents the observed agreement and Pe denotes the expected agreement by chance, resulted in values of 0.65 for significant fibrosis and 0.72 for advanced fibrosis.

**Clinical parameters**

The formulas for FibroScan-aspartate aminotransferase (FAST), Agile 3+, aspartate aminotransferase (AST) to platelet (PLT) ratio index (APRI), and fibrosis-4 index (FIB-4) involved in this study were calculated according to the following formula:^3-5^

$$FAST=\frac{e^{-1.65+1.07\times ln\left（ \mathrm{LSM} \right）+2.66*{10}^{-8}}\times\mathrm{CAP}^{3}-63.3\times\mathrm{AST}^{-1}}{1+e^{-1.65+1.07\times ln\left（ \mathrm{LSM} \right）+2.66*{10}^{-8}}}$$

$$Agile 3+=\frac{e^{{logit(}_{P_{F}\geq3})}}{1+e^{{logit(}_{P_{F}\geq3})}}$$

$$APRI=\frac{AST/(AST (Upper limit of normal))}{\mathrm{PLT}}\times100$$

$$FIB-4=\frac{Age\times AST}{PLT\times\sqrt{\mathrm{ALT}}}$$

Note: ${logit(}_{P_{F}\geq3})=-3.92368+2.29714\times\ln(LSM)-0.00902\times PLT－0.98633\times\mathrm{AAR}^{-1}+1.08636\times Diabetes status－0.38581\times\mathrm{Gender}+0.03018\times\mathrm{Age}$; AAR=AST/ALT; LSM, liver stiffness measurement; CAP, controlled attenuation parameter; ALT, alanine aminotransferase.

**References**

1. Rui F, Xu L, Yeo YH, et al. Machine Learning-Based Models for Advanced Fibrosis and Cirrhosis Diagnosis in Chronic Hepatitis B Patients With Hepatic Steatosis. *Clin Gastroenterol Hepatol* 2024; 22(11): 2250-60.e12.

2. Landis JR, Koch GG. The measurement of observer agreement for categorical data. *Biometrics* 1977; 33(1): 159-74.

3. Newsome PN, Sasso M, Deeks JJ, et al. FibroScan-AST (FAST) score for the non-invasive identification of patients with non-alcoholic steatohepatitis with significant activity and fibrosis: a prospective derivation and global validation study. *Lancet Gastroenterol Hepatol* 2020; 5(4): 362-73.

4. Dalbeni A, Lombardi R, Henrique M, et al. Diagnostic accuracy of AGILE 3+ score for advanced fibrosis in patients with NAFLD: A systematic review and meta-analysis. *Hepatology* 2024; 79(5): 1107-16.

5. Itakura J, Kurosaki M, Setoyama H, et al. Applicability of APRI and FIB-4 as a transition indicator of liver fibrosis in patients with chronic viral hepatitis. *J Gastroenterol* 2021; 56(5): 470-8.

**Supplementary Table 1.** Baseline characteristics of CHB patients with available LSM stratified according to liver fibrosis stages

| Variable | Overall  n = 167 | S0-1  n = 77 | S2-4  n = 90 | *P*-value | S0-2  n = 117 | S3-4  n = 50 | *P*-value |
| --- | --- | --- | --- | --- | --- | --- | --- |
| Male, n (%) | 107 (64.1%) | 52 (67.5%) | 55 (61.1%) | 0.484 | 75 (64.1%) | 32 (64.0%) | 1.000 |
| Age (years) | 39.0 (33.0, 46.0) | 40.0 (33.0, 47.0) | 38.5 (33.0, 45.0) | 0.817 | 40.0 (34.0, 46.0) | 38.0 (33.0, 45.0) | 0.440 |
| BMI (kg/m^2^) | 23.44 (21.61, 25.54) | 23.03 (21.22, 24.91) | 23.96 (22.10, 25.77) | 0.193 | 23.20 (21.67, 25.47) | 24.11 (21.48, 25.57) | 0.800 |
| WBC (10^9^/L) | 5.0 (4.4, 6.0) | 5.1 (4.5, 6.0) | 5.0 (4.3, 6.1) | 0.466 | 5.2 (4.5, 6.3) | 4.7 (4.1, 5.5) | 0.013 |
| Hb (g/L) | 145 (134, 158) | 144 (133, 152) | 147 (137, 161) | 0.096 | 144 (134, 153) | 151 (138, 164) | 0.051 |
| PLT (10^9^/L) | 166 (139, 206) | 179 (156, 215) | 160 (120, 184) | 0.003 | 169 (149, 213) | 140 (106, 177) | 0.003 |
| TBIL (umol/L) | 13.1 (10.1, 17.9) | 12.7 (9.0, 17.8) | 13.5 (10.7, 17.8) | 0.166 | 12.7 (9.0, 16.9) | 15.7 (11.6, 20.2) | 0.010 |
| ALB (g/L) | 42.4 (38.7, 45.6) | 41.8 (37.9, 44.1) | 42.9 (39.8, 46.2) | 0.016 | 41.8 (38.7, 44.1) | 44.7 (39.0, 47.5) | 0.005 |
| ALT (U/L) | 38.5 (25.0, 62.9) | 34.0 (20.4, 55.4) | 43.0 (27.8, 87.6) | 0.010 | 37.6 (24.1, 59.9) | 43.0 (28.3, 105.9) | 0.065 |
| AST (U/L) | 26.0 (20.0, 38.0) | 21.0 (19.0, 28.1) | 28.0 (23.0, 46.8) | <0.001 | 23.0 (19.0, 33.0) | 30.0 (24.3, 50.3) | <0.001 |
| ALP (U/L) | 63.0 (49.3, 80.5) | 57.0 (42.1, 67.0) | 70.4 (56.2, 87.4) | <0.001 | 60.1 (45.6, 74.0) | 73.5 (57.5, 88.5) | <0.001 |
| GGT (U/L) | 29.0 (17.5, 44.2) | 21.7 (16.2, 40.4) | 37.9 (21.0, 69.0) | <0.001 | 27.0 (17.0, 40.7) | 41.5 (23.0, 84.1) | 0.001 |
| HBsAg (IU/mL) | 3158 (848, 9199) | 3064 (622, 13970) | 3247 (1395, 7787) | 0.898 | 3446 (811, 12868) | 2948 (1395, 5267) | 0.414 |
| HBeAg positive, n (%) | 73 (43.7%) | 22 (28.6%) | 51 (56.7%) | <0.001 | 45 (38.5%) | 28 (56.0%) | 0.055 |
| HBV DNA (log IU/mL) | 4 (2, 6) | 3 (0, 5) | 4.5 (3.0, 6.0) | <0.001 | 3 (0, 7) | 5 (3, 6) | 0.014 |
| PRO-C3 (ng/mL) | 27.56 (12.80, 50.55) | 15.07 (10.56, 26.57) | 47.45 (25.58, 63.26) | <0.001 | 17.80 (11.92, 35.70) | 50.58 (37.28, 68.93) | <0.001 |
| GP73 (ng/mL) | 24.98 (17.93, 38.35) | 18.99 (14.42, 26.83) | 30.94 (21.04, 54.10) | <0.001 | 19.98 (16.09, 32.35) | 33.31 (22.22, 64.91) | <0.001 |
| LSM (kpa) | 7.4 (5.9, 11.4) | 6.4 (5.3, 7.9) | 10.3 (6.4, 14.1) | <0.001 | 6.4 (5.3, 8.6) | 11.6 (8.5, 15.0) | <0.001 |
| FAST | 0.19 (0.08, 0.35) | 0.10 (0.05, 0.22) | 0.27 (0.12, 0.52) | <0.001 | 0.11 (0.06, 0.27) | 0.34 (0.19, 0.57) | <0.001 |
| Agile 3+ | 0.23 (0.08, 0.47) | 0.13 (0.06, 0.28) | 0.38 (0.14, 0.70) | <0.001 | 0.14 (0.06, 0.33) | 0.46 (0.25, 0.79) | <0.001 |
| FIB-4 | 1.08 (0.76, 1.57) | 0.94 (0.69, 1.32) | 1.17 (0.82, 1.95) | 0.003 | 0.96 (0.74, 1.36) | 1.31 (0.92, 2.30) | 0.002 |
| APRI | 0.4 (0.3, 0.7) | 0.3 (0.2, 0.4) | 0.5 (0.4, 0.8) | <0.001 | 0.3 (0.2, 0.5) | 0.7 (0.4, 0.9) | <0.001 |

Note: *P*-values, Mann-Whitney U test for continuous variables; Chi-squared test for qualitative variables. Abbreviation: CHB, chronic hepatitis B; LSM, liver stiffness measurement; BMI, body mass index; WBC, white blood cell; Hb, hemoglobin; PLT, platelet; TBIL, total bilirubin; ALB, albumin; ALT, alanine aminotransferase; AST, aspartate aminotransferase; ALP, alkaline phosphatase; GGT, gamma-glutamyltransferase; HBsAg, hepatitis B surface antigen; HBeAg, hepatitis B e-antigen; PRO‑C3, N-terminal propeptide of type III collagen; GP73, Golgi protein 73; S, fibrosis stage; FAST, FibroScan-aspartate aminotransferase; FIB-4, fibrosis-4 index; APRI, aspartate aminotransferase‑to‑platelet ratio index.

**Supplementary Table 2.** Diagnostic accuracy of different NITs for significant fibrosis (S2-4) and advanced fibrosis (S3-4) in CHB patients

| NITs | AUC (95% CI) | PPV (95% CI) | NPV (95% CI) | Sensitivity (95% CI) | | Specificity (95% CI) |
| --- | --- | --- | --- | --- | --- | --- |
| Significant fibrosis (S2-4) | | | | | | |
| PRO-C3 (ng/mL) | 0.81 (0.74-0.87) | 0.89 (0.79-0.94) | 0.67 (0.61-0.72) | 0.61 (0.50-0.71) | 0.91 (0.82-0.96) | |
| GP73 (ng/mL) | 0.75 (0.68-0.83) | 0.74 (0.66-0.81) | 0.66 (0.58-0.73) | 0.68 (0.57-0.77) | 0.73 (0.61-0.82) | |
| PRO-C3+GP73 | 0.84 (0.78-0.89) | 0.83 (0.75-0.89) | 0.71 (0.64-0.78) | 0.71 (0.61-0.80) | 0.83 (0.73-0.91) | |
| LSM (kpa) | 0.74 (0.67-0.82) | 0.91 (0.81-0.96) | 0.64 (0.59-0.70) | 0.56 (0.45-0.66) | 0.94 (0.86-0.98) | |
| FAST | 0.72 (0.65-0.80) | 0.69 (0.63-0.75) | 0.70 (0.60-0.79) | 0.79 (0.69-0.87) | 0.58 (0.47-0.70) | |
| Agile 3+ | 0.72 (0.65-0.80) | 0.80 (0.70-0.87) | 0.63 (0.57-0.69) | 0.58 (0.47-0.68) | 0.83 (0.73-0.91) | |
| FIB-4 | 0.64 (0.55-0.72) | 0.79 (0.64-0.89) | 0.53 (0.49-0.56) | 0.30 (0.21-0.41) | 0.91 (0.82-0.96) | |
| APRI | 0.72 (0.64-0.79) | 0.69 (0.63-0.75) | 0.68 (0.59-0.76) | 0.76 (0.65-0.84) | 0.61 (0.49-0.72) | |
| Advanced fibrosis (S3-4) | | | | | | |
| PRO-C3 (ng/mL) | 0.80 (0.73-0.87) | 0.62 (0.52-0.71) | 0.87 (0.81-0.91) | 0.72 (0.58-0.84) | 0.81 (0.73-0.88) | |
| GP73 (ng/mL) | 0.73 (0.65-0.81) | 0.48 (0.41-0.56) | 0.86 (0.79-0.91) | 0.74 (0.60-0.85) | 0.66 (0.57-0.74) | |
| PRO-C3+GP73 | 0.80 (0.73-0.86) | 0.62 (0.52-0.71) | 0.87 (0.81-0.91) | 0.72 (0.58-0.84) | 0.81 (0.73-0.88) | |
| LSM (kpa) | 0.83 (0.77-0.90) | 0.52 (0.45-0.58) | 0.94 (0.87-0.97) | 0.90 (0.78-0.97) | 0.64 (0.55-0.73) | |
| FAST | 0.76 (0.68-0.84) | 0.46 (0.40-0.52) | 0.90 (0.82-0.95) | 0.86 (0.73-0.94) | 0.56 (0.47-0.66) | |
| Agile 3+ | 0.79 (0.72-0.87) | 0.56 (0.47-0.65) | 0.86 (0.80-0.91) | 0.72 (0.58-0.84) | 0.76 (0.67-0.84) | |
| FIB-4 | 0.65 (0.56-0.75) | 0.57 (0.43-0.70) | 0.78 (0.73-0.82) | 0.42 (0.28-0.57) | 0.86 (0.79-0.92) | |
| APRI | 0.72 (0.64-0.81) | 0.58 (0.47-0.68) | 0.83 (0.77-0.87) | 0.60 (0.45-0.74) | 0.81 (0.73-0.88) | |

Note: Logistic regression was performed to calculate predicted values of PRO-C3+GP73. Abbreviation: NITs: non-invasive tests; CHB, chronic hepatitis B; S, fibrosis stage; PRO-C3, N-terminal propeptide of type III collagen; GP73, Golgi protein 73; LSM, liver stiffness measurement; FAST, FibroScan-aspartate aminotransferase; FIB-4, fibrosis-4 index; AUC, area under the curve; PPV, positive predictive value; NPV, negative predictive value; APRI, aspartate aminotransferase‑to‑platelet ratio index.

**Supplementary Table 3.** Multivariate logistic regression analysis of PRO-C3 of significant fibrosis (S2-4) and advanced fibrosis (S3-4) under different subgroups by gender, age (years), BMI (kg/m^2^), ALT (U/L), steatosis, HBeAg and HBV DNA (log IU/mL)

| Subgroups | Significant fibrosis (S2-4) | | Advanced fibrosis (S3-4) | |
| --- | --- | --- | --- | --- |
|  | aOR (95% CI) | *P*-value | aOR (95% CI) | *P*-value |
| (A) | | | | |
| Male | 4.90 (2.46-9.74) | <0.001^a1^ | 16.75 (6.34-44.98) | <0.001^a2^ |
| Female | 3.96 (1.65-9.51) | <0.01^a3^ | 6.59 (2.45-17.70) | <0.01^a4^ |
| (B) | | | | |
| Age < 40 years | 5.17 (2.38-11.24) | <0.001^b1^ | 18.25 (5.69-58.52) | <0.001^b2^ |
| Age ≥ 40 years | 3.91 (1.84-8.31) | <0.001^b3^ | / | 0.28^b4^ |
| (C) | | | | |
| BMI < 23 kg/m^2^ | 13.81 (4.50-42.33) | <0.001^c1^ | 7.44 (2.69-20.58) | <0.001^c2^ |
| BMI ≥ 23 kg/m^2^ | 6.48 (3.17-13.25) | <0.001^c3^ | 11.54 (4.35-30.63) | <0.001^c4^ |
| (D) | | | | |
| ALT normal | 6.92 (2.93-16.33) | <0.001^d1^ | 11.16 (3.44-36.21) | <0.001^d2^ |
| ALT elevation | 5.51 (2.59-11.71) | <0.001^d3^ | 7.06 (2.97-16.79) | <0.001^d4^ |
| (E) | | | | |
| Without steatosis | 4.92 (2.51-9.65) | <0.001^e1^ | 6.07 (2.50-14.73) | <0.001^e2^ |
| With steatosis | 5.51 (2.11-14.41) | <0.01^e3^ | 15.06 (4.32-52.48) | <0.001^e4^ |
| (F) | | | | |
| HBeAg negative | 4.38 (2.22-8.67) | <0.001^f1^ | 14.19 (5.51-36.59) | <0.001^f2^ |
| HBeAg positive | 4.62 (1.73-12.29) | <0.01^f3^ | 8.21 (2.25-29.96) | <0.001^f4^ |
| (G) | | | | |
| HBV DNA < 4 log IU/mL | 4.47 (2.01-9.93) | <0.001^g1^ | 35.34 (9.43-132.46) | <0.001^g2^ |
| HBV DNA ≥ 4 log IU/mL | 5.19 (2.47-10.92) | <0.001^g3^ | 3.64 (1.71-7.75) | <0.01^g4^ |

Note: (A) adjusted for GP73, age, BMI, ALT, AST, ALP, GGT, HBeAg and HBV DNA. (B) adjusted for GP73, gender, BMI, ALT, AST, ALP, GGT, HBeAg and HBV DNA. (C) adjusted for GP73, gender, age, ALT, AST, ALP, GGT, HBeAg and HBV DNA. (D) adjusted for GP73, gender, age, BMI, AST, ALP, GGT, HBeAg and HBV DNA. (E) adjusted for GP73, gender, age, BMI, ALT, AST, ALP, GGT, HBeAg and HBV DNA. (F) adjusted for GP73, gender, age, BMI, ALT, AST, ALP, GGT, and HBV DNA. (G) adjusted for GP73, gender, age, BMI, ALT, AST, ALP, GGT, and HBeAg. PRO-C3 was transformed into categorical variables based on Youden Index-derived cutoff values within each subgroup. a1, PRO-C3 >27.66 ng/mL; a2, PRO-C3 >41.61 ng/mL; a3, PRO-C3 >28.95 ng/mL; a4, PRO-C3 >28.95 ng/mL; b1, PRO-C3 >28.95 ng/mL; b2, PRO-C3 >39.15 ng/mL; b3, PRO-C3 >30.35 ng/mL; b4, PRO-C3 >34.57 ng/mL; c1, PRO-C3 >35.70 ng/mL; c2, PRO-C3> 33.75 ng/mL; c3, PRO-C3 >27.66 ng/mL; c4, PRO-C3 >42.32 ng/mL; d1, PRO-C3 >32.51 ng/mL; d2, PRO-C3 >37.85 ng/mL; d3, PRO-C3 >35.70 ng/mL; d4, PRO-C3 >35.70 ng/mL; e1, PRO-C3 >28.95 ng/mL; e2, PRO-C3 >26.57 ng/mL; e3, PRO-C3 >37.85 ng/mL; e4, PRO-C3 >39.04 ng/mL; f1, PRO-C3 >28.95 ng/mL; f2, PRO-C3 >44.55 ng/mL; f3, PRO-C3 >25.83 ng/mL; f4, PRO-C3 >35.32 ng/mL; g1, PRO-C3 >30.25 ng/mL; g2, PRO-C3 >35.7 ng/mL; g3, PRO-C3 >27.93 ng/mL; g4, PRO-C3 >42.32 ng/mL. ALT normal: ALT ≤35 U/L in the male, ALT ≤25 U/L in the female; ALT elevation: ALT >35 U/L in the male, ALT >25 U/L in the female. Abbreviation: PRO-C3, N-terminal propeptide of type III collagen; S, fibrosis stage; BMI, body mass index; aOR: adjusted odds ratio; ALT, alanine aminotransferase; HBeAg, hepatitis B e-antigen; AST, aspartate aminotransferase; ALP, alkaline phosphatase; GGT, gamma-glutamyltransferase.

**Supplementary Table 4.** Multivariate logistic regression analysis of GP73 of significant fibrosis (S2-4) and advanced fibrosis (S3-4) under different subgroups by gender, age (years), BMI (kg/m^2^), ALT (U/L), steatosis, HBeAg and HBV DNA (log IU/mL)

| Subgroups | Significant fibrosis (S2-4) | | Advanced fibrosis (S3-4) | |
| --- | --- | --- | --- | --- |
|  | OR (95% CI) | *P*-value | OR (95% CI) | *P*-value |
| (A) | | | | |
| Male | 3.26 (1.63-6.54) | <0.01^a1^ | 5.82 (2.38-14.23) | <0.001^a2^ |
| Female | 3.91 (1.52-10.04) | 0.01^a3^ | / | 0.10^a4^ |
| (B) | | | | |
| Age < 40 years | 3.84 (1.71-8.62) | <0.01^b1^ | 5.68 (2.07-15.56) | <0.01^b2^ |
| Age ≥ 40 years | 3.22 (1.53-6.80) | <0.01^b3^ | 5.61 (2.46-12.81) | <0.001^b4^ |
| (C) | | | | |
| BMI < 23 kg/m^2^ | 3.86 (1.50-9.95) | 0.01^c1^ | 4.38 (1.26-15.19) | 0.02^c2^ |
| BMI ≥ 23 kg/m^2^ | 4.72 (2.33-9.54) | <0.001^c3^ | 5.21 (2.25-12.07) | <0.001^c4^ |
| (D) | | | | |
| ALT normal | / | 0.08^d1^ | 5.74 (1.86-17.75) | <0.01^d2^ |
| ALT elevation | 4.01 (1.93-8.37) | 0.07^d3^ | 3.39 (1.49-7.67) | <0.01^d4^ |
| (E) | | | | |
| Without steatosis | 2.15 (1.01-4.56) | 0.047^e1^ | 3.78 (1.69-8.45) | <0.01^e2^ |
| With steatosis | 8.26 (3.36-20.30) | <0.001^e3^ | 11.90 (2.99-47.37) | <0.001^e4^ |
| (F) | | | | |
| HBeAg negative | 3.69 (1.80-7.56) | <0.001^f1^ | 3.10 (1.27-7.58) | 0.01^f2^ |
| HBeAg positive | 2.91 (1.10-7.70) | 0.03^f3^ | 20.88 (4.69-93.04) | <0.01^f4^ |
| (G) | | | | |
| HBV DNA < 4 log IU/mL | 3.16 (1.39-7.19) | 0.01^g3^ | 9.07 (2.56-32.16) | <0.01^g2^ |
| HBV DNA ≥ 4 log IU/mL | 4.11 (1.93-8.79) | <0.001^g3^ | 3.84 (1.78-8.30) | <0.01^g4^ |

Note: (A) adjusted for GP73, age, BMI, ALT, AST, ALP, GGT, HBeAg and HBV DNA. (B) adjusted for GP73, gender, BMI, ALT, AST, ALP, GGT, HBeAg and HBV DNA. (C) adjusted for GP73, gender, age, ALT, AST, ALP, GGT, HBeAg and HBV DNA. (D) adjusted for GP73, gender, age, BMI, AST, ALP, GGT, HBeAg and HBV DNA. (E) adjusted for GP73, gender, age, BMI, ALT, AST, ALP, GGT, HBeAg and HBV DNA. (F) adjusted for GP73, gender, age, BMI, ALT, AST, ALP, GGT, and HBV DNA. (G) adjusted for GP73, gender, age, BMI, ALT, AST, ALP, GGT, and HBeAg. PRO-C3 was transformed into categorical variables based on Youden Index-derived cutoff values within each subgroup. a1, GP73 >23.07 ng/mL; a2, GP73 >25.05 ng/mL; a3, GP73 >28.99 ng/mL; a4, GP73 >28.99 ng/mL; b1, GP73 >25.67 ng/mL; b2, GP73 >28.99 ng/mL; b3, GP73 >22.71 ng/mL; b4, GP73 >19.59 ng/mL; c1, GP73 >18.20 ng/mL; c2, GP73 >19.59 ng/mL; c3, GP73 >23.76 ng/mL; c4, GP73 >28.99 ng/mL; d1, GP73 >18.99 ng/mL; d2, GP73 >25.05 ng/mL; d3, GP73 >5.24 ng/mL; d4, GP73 >28.99 ng/mL; e1, GP73 >28.99 ng/mL; e2, GP73 >28.99 ng/mL; e3, GP73 >24.21 ng/mL; e4, GP73 >24.98 ng/mL; f1, GP73 >18.99 ng/mL; f2, GP73 >24.99 ng/mL; f3, GP73 >24.21 ng/mL; f4, GP73 >28.79 ng/mL; g1, GP73 >18.99 ng/mL; g2, GP73 >25.05 ng/mL; g3, GP73 >24.21 ng/mL; g4, GP73 >29.52 ng/mL. ALT normal: ALT ≤35 U/L in the male, ALT ≤25 U/L in the female; ALT elevation: ALT >35 U/L in the male, ALT >25 U/L in the female. Abbreviation: GP73, Golgi protein 73; S, fibrosis stage; BMI, body mass index; aOR: adjusted odds ratio; ALT, alanine aminotransferase; HBeAg, hepatitis B e-antigen; AST, aspartate aminotransferase; ALP, alkaline phosphatase; GGT, gamma-glutamyltransferase.

**Supplementary Table 5.** Diagnostic accuracy of different NITs for significant fibrosis under different subgroups by gender, age (years), BMI (kg/m^2^), ALT (U/L), steatosis, HBeAg and HBV DNA (log IU/mL)

| Subgroups | NITs | AUC (95% CI) | PPV (95% CI) | NPV (95% CI) | Sensitivity (95% CI) | Specificity (95% CI) |
| --- | --- | --- | --- | --- | --- | --- |
| Male | | | | | | |
|  | PRO-C3 (ng/mL) | 0.81 (0.73-0.90) | 0.80 (0.69-0.88) | 0.74 (0.64-0.82) | 0.73 (0.59-0.84) | 0.81 (0.68-0.90) |
|  | GP73 (ng/mL) | 0.78 (0.69-0.87) | 0.77 (0.66-0.85) | 0.71 (0.62-0.80) | 0.71 (0.57-0.82) | 0.77 (0.63-0.88) |
|  | PRO-C3+GP73 | 0.85 (0.78-0.92) | 0.77 (0.67-0.84) | 0.77 (0.66-0.85) | 0.78 (0.65-0.88) | 0.75 (0.61-0.86) |
|  | LSM (kpa) | 0.79 (0.70-0.88) | 0.90 (0.77-0.96) | 0.71 (0.63-0.77) | 0.64 (0.50-0.76) | 0.92 (0.82-0.98) |
|  | FAST | 0.74 (0.65-0.84) | 0.67 (0.60-0.73) | 0.84 (0.69-0.93) | 0.91 (0.80-0.97) | 0.52 (0.38-0.66) |
|  | Agile 3+ | 0.76 (0.66-0.85) | 0.85 (073-0.93) | 0.70 (0.62-0.77) | 0.64 (0.50-0.76) | 0.88 (0.77-0.96) |
|  | FIB-4 | 0.66 (0.56-0.76) | 0.64 (0.54-0.73) | 0.61 (0.51-0.70) | 0.62 (0.48-0.75) | 0.64 (0.49-0.76) |
|  | APRI | 0.72 (0.63-0.82) | 0.71 (0.61-0.80) | 0.66 (0.56-0.74) | 0.64 (0.50-0.65) | 0.83 (0.70-0.92) |
| Female | | | | | | |
|  | PRO-C3 (ng/mL) | 0.79 (0.68-0.91) | 0.96 (0.76-0.99) | 0.65 (0.54-0.74) | 0.63 (0.45-0.79) | 0.96 (0.80-1.00) |
|  | GP73 (ng/mL) | 0.70 (0.56-0.83) | 0.79 (0.62-0.90) | 0.56 (0.45-0.65) | 0.54 (0.37-0.71) | 0.80 (0.59-0.93) |
|  | PRO-C3+GP73 | 0.79 (0.68-0.91) | 0.96 (0.76-0.99) | 0.65 (0.54-0.74) | 0.63 (0.45-0.79) | 0.96 (0.80-1.00) |
|  | LSM (kpa) | 0.69 (0.56-0.82) | 0.94 (0.68-0.99) | 0.55 (0.47-0.62) | 0.43 (0.26-0.61) | 0.96 (0.80-1.00) |
|  | FAST | 0.73 (0.60-0.86) | 0.90 (0.68-0.97) | 0.56 (0.48-0.64) | 0.49 (0.31-0.66) | 0.92 (0.74-0.99) |
|  | Agile 3+ | 0.65 (0.52-0.79) | 0.69 (0.58-0.79) | 0.58 (0.43-0.72) | 0.71 (0.54-0.85) | 0.56 (0.35-0.76) |
|  | FIB-4 | 0.60 (0.46-0.75) | 0.85 (0.57-0.96) | 0.49 (0.43-0.55) | 0.31 (0.17-0.49) | 0.92 (0.74-0.99) |
|  | APRI | 0.73 (0.61-0.86) | 0.79 (0.65-0.89) | 0.61 (0.49-0.73) | 0.66 (0.48-0.81) | 0.76 (0.55-0.91) |
| Age < 40 years | | | | | | |
|  | PRO-C3 (ng/mL) | 0.83 (0.73-0.92) | 0.87 (0.75-0.94) | 0.70 (0.60-0.79) | 0.71 (0.56-0.83) | 0.87 (0.72-0.96) |
|  | GP73 (ng/mL) | 0.79 (0.69-0.88) | 0.83 (0.70-0.90) | 0.67 (0.57-0.76) | 0.69 (0.54-0.81) | 0.82 (0.66-0.92) |
|  | PRO-C3+GP73 | 0.87 (0.80-0.95) | 0.86 (0.74-0.93) | 0.74 (0.63-0.83) | 0.77 (0.63-0.88) | 0.84 (0.69-0.94) |
|  | LSM (kpa) | 0.72 (0.61-0.83) | 0.88 (0.73-0.95) | 0.63 (0.55-0.71) | 0.58 (0.43-0.72) | 0.90 (0.75-0.97) |
|  | FAST | 0.72 (0.60 -0.83) | 0.74 (0.64-0.81) | 0.73 (0.59-0.83) | 0.81 (0.67-0.91) | 0.63 (0.46-0.78) |
|  | Agile 3+ | 0.76 (0.66-0.86) | 0.85 (0.71-0.93) | 0.64 (0.55-0.72) | 0.60 (0.45-0.74) | 0.87 (0.72-0.96) |
|  | FIB-4 | 0.71 (0.60-0.82) | 0.72 (0.62-0.81) | 0.64 (0.52-0.75) | 0.71 (0.56-0.83) | 0.66 (0.49-0.80) |
|  | APRI | 0.71 (0.59-0.82) | 0.70 (0.61-0.78) | 0.67 (0.53-0.78) | 0.77 (0.63-0.88) | 0.58 (0.41-0.74) |
| Age ≥ 40 years | | | | | | |
|  | PRO-C3 (ng/mL) | 0.78 (0.69-0.88) | 0.91 (0.73-0.98) | 0.69 (0.56-0.71) | 0.50 (0.34-0.66) | 0.95 (0.83-0.99) |
|  | GP73 (ng/mL) | 0.71 (0.60-0.82) | 0.68 (0.58-0.77) | 0.71 (0.57-0.81) | 0.76 (0.61-0.88) | 0.62 (0.45-0.77) |
|  | PRO-C3+GP73 | 0.78 (0.69-0.88) | 0.91 (0.70-0.97) | 0.62 (0.55-0.68) | 0.45 (0.30-0.61) | 0.95 (0.83-0.99) |
|  | LSM (kpa) | 0.77 (0.66-0.87) | 0.92 (0.75-0.98) | 0.67 (0.59-0.75) | 0.57 (0.41-0.72) | 0.95 (0.83-0.99) |
|  | FAST | 0.74 (0.63-0.85) | 0.79 (0.66-0.89) | 0.68 (0.58-0.77) | 0.64 (0.48-0.78) | 0.82 (0.67-0.93) |
|  | Agile 3+ | 0.75 (0.64-0.86) | 0.96 (0.76-0.99) | 0.66 (0.58-0.72) | 0.52 (0.36-0.68) | 0.97 (0.87-1.00) |
|  | FIB-4 | 0.67 (0.55-0.79) | 0.90 (0.69-0.97) | 0.61 (0.54-0.67) | 0.43 (0.28-0.59) | 0.95 (0.83-0.99) |
|  | APRI | 0.73 (0.61-0.84) | 0.69 (0.58-0.78) | 0.69 (0.57-0.80) | 0.74 (0.58-0.86) | 0.64 (0.47-0.79) |
| BMI < 23 kg/m^2^ | | | | | | |
|  | PRO-C3 (ng/mL) | 0.85 (0.76-0.94) | 0.74 (0.62-0.83) | 0.82 (0.69-0.91) | 0.82 (0.66-0.93) | 0.74 (0.56-0.87) |
|  | GP73 (ng/mL) | 0.69 (0.56-0.81) | 0.61 (0.51-0.71) | 0.71 (0.57-0.82) | 0.74 (0.56-0.87) | 0.58 (0.41-0.74) |
|  | PRO-C3+GP73 | 0.85 (0.76-0.94) | 0.74 (0.62-0.83) | 0.82 (0.69-0.91) | 0.82 (0.66-0.93) | 0.74 (0.57-0.87) |
|  | LSM (kpa) | 0.71 (0.59-0.84) | N.A | 0.67 (0.60-0.73) | 0.44 (0.27-0.62) | 1.00 (0.91-1.00) |
|  | FAST | 0.74 (0.62-0.86) | 0.68 (0.56-0.78) | 0.74 (0.61-0.84) | 0.74 (0.56-0.87) | 0.68 (0.51-0.83) |
|  | Agile 3+ | 0.70 (0.58-0.83) | 0.68 (0.55-0.78) | 0.71 (0.59-0.81) | 0.68 (0.50-0.83) | 0.71 (0.54-0.85) |
|  | FIB-4 | 0.70 (0.58-0.82) | 0.67 (0.53-0.78) | 0.69 (0.58-0.79) | 0.65 (0.47-0.80) | 0.71 (0.54-0.85) |
|  | APRI | 0.75 (0.63-0.86) | 0.74 (0.58-0.86) | 0.69 (0.59-0.77) | 0.59 (0.41-0.75) | 0.82 (0.66-0.92) |
| BMI ≥ 23 kg/m^2^ | | | | | | |
|  | PRO-C3 (ng/mL) | 0.77 (0.67-0.87) | 0.86 (0.74-0.93) | 0.63 (0.53-0.71) | 0.64 (0.50-0.77) | 0.85 (0.70-0.94) |
|  | GP73 (ng/mL) | 0.80 (0.71-0.89) | 0.81 (0.71-0.88) | 0.67 (0.56-0.77) | 0.75 (0.62-0.86) | 0.74 (0.58-0.87) |
|  | PRO-C3+GP73 | 0.83 (0.75-0.91) | 0.80 (0.71-0.86) | 0.75 (0.61-0.85) | 0.84 (0.72-0.92) | 0.69 (0.52-0.83) |
|  | LSM (kpa) | 0.75 (0.65-0.85) | 0.88 (0.75-0.94) | 0.62 (0.53-0.70) | 0.63 (0.49-0.75) | 0.87 (0.73-0.96) |
|  | FAST | 0.69 (0.58-0.80) | 0.74 (0.64-0.81) | 0.60 (0.48-0.70) | 0.70 (0.56-0.81) | 0.64 (0.47-0.79) |
|  | Agile 3+ | 0.73 (0.63-0.83) | 0.82 (0.70-0.90) | 0.61 (0.51-0.70) | 0.64 (0.50-0.77) | 0.79 (0.64-0.91) |
|  | FIB-4 | 0.57 (0.46-0.69) | 0.93 (0.66-0.99) | 0.48 (0.44-0.52) | 0.25 (0.14-0.38) | 0.97 (0.87-1.00) |
|  | APRI | 0.69 (0.58-0.80) | 0.72 (0.63-0.79) | 0.63 (0.49-0.75) | 0.77 (0.64-0.87) | 0.56 (0.40-0.72) |
| ALT normal | | | | | | |
|  | PRO-C3 (ng/mL) | 0.83 (0.72-0.93) | 0.83 (0.66-0.93) | 0.80 (0.69-0.88) | 0.71 (0.51-0.87) | 0.89 (0.74-0.97) |
|  | GP73 (ng/mL) | 0.67 (0.53-0.80) | 0.60 (0.47-0.72) | 0.71 (0.47-0.72) | 0.64 (0.44-0.81) | 0.67 (0.49-0.81) |
|  | PRO-C3+GP73 | 0.83 (0.72-0.93) | 0.83 (0.66-0.93) | 0.80 (0.69-0.88) | 0.71 (0.51-0.87) | 0.89 (0.74-0.97) |
|  | LSM (kpa) | 0.71 (0.58-0.85) | 0.93 (0.64-0.99) | 0.70 (0.62-0.77) | 0.46 (0.28-0.66) | 0.97 (0.86-1.00) |
|  | FAST | 0.72 (0.58-0.86) | 0.76 (0.57-0.89) | 0.72 (0.62-0.80) | 0.57 (0.37-0.76) | 0.86 (0.71-0.95) |
|  | Agile 3+ | 0.77 (0.66-0.89) | 0.71 (0.54-0.83) | 0.73 (0.62-0.81) | 0.61 (0.41-0.79) | 0.81 (0.64-0.92) |
|  | FIB-4 | 0.65 (0.51-0.79) | 0.59 (0.45-0.72) | 0.68 (0.56-0.77) | 0.57 (0.37-0.76) | 0.69 (0.52-0.84) |
|  | APRI | 0.73 (0.60-0.86) | 0.76 (0.57-0.89) | 0.72 (0.62-0.80) | 0.57 (0.37-0.76) | 0.86 (0.71-0.95) |
| ALT elevation | | | | | | |
|  | PRO-C3 (ng/mL) | 0.79 (0.70-0.88) | 0.81 (0.72-0.88) | 0.68 (0.57-0.78) | 0.77 (0.65-0.87) | 0.73 (0.57-0.86) |
|  | GP73 (ng/mL) | 0.80 (0.72-0.89) | 0.83 (0.73-0.90) | 0.63 (0.52-0.72) | 0.71 (0.58-0.82) | 0.78 (0.62-0.89) |
|  | PRO-C3+GP73 | 0.85 (0.78-0.92) | 0.84 (0.74-0.90) | 0.67 (0.56-0.76) | 0.74 (0.62-0.85) | 0.78 (0.62-0.89) |
|  | LSM (kpa) | 0.75 (0.65-0.84) | 0.91 (0.79-0.96) | 0.61 (0.53-0.68) | 0.61 (0.48-0.73) | 0.90 (0.77-0.97) |
|  | FAST | 0.70 (0.60-0.80) | 0.81 (0.68-0.89) | 0.53 (0.46-0.61) | 0.53 (0.40-0.66) | 0.80 (0.65-0.91) |
|  | Agile 3+ | 0.73 (0.64-0.83) | 0.90 (0.77-0.96) | 0.57 (0.50-0.64) | 0.55 (0.42-0.68) | 0.90 (0.77-0.97) |
|  | FIB-4 | 0.65 (0.54-0.75) | 0.70 (0.62-0.77) | 0.55 (0.43-0.66) | 0.71 (0.58-0.82) | 0.54 (0.37-0.69) |
|  | APRI | 0.69 (0.58-0.79) | 0.79 (0.68-0.87) | 0.55 (0.47-0.64) | 0.60 (0.46-0.72) | 0.76 (0.60-0.88) |
| Without steatosis | | | | | | |
|  | PRO-C3 (ng/mL) | 0.86 (0.79-0.93) | 0.88 (0.78-0.94) | 0.76 (0.67-0.83) | 0.75 (0.62-0.85) | 0.89 (0.77-0.96) |
|  | GP73 (ng/mL) | 0.72 (0.63-0.82) | 0.70 (0.60-0.79) | 0.63 (0.54-0.71) | 0.63 (0.50-0.75) | 0.70 (0.56-0.82) |
|  | PRO-C3+GP73 | 0.88 (0.81-0.94) | 0.85 (0.75-0.91) | 0.82 (0.71-0.89) | 0.83 (0.72-0.92) | 0.83 (0.70-0.92) |
|  | LSM (kpa) | 0.76 (0.68-0.85) | 0.94 (0.81-0.99) | 0.65 (0.59-0.72) | 0.55 (0.42-0.68) | 0.96 (0.87-1.00) |
|  | FAST | 0.77 (0.68-0.86) | 0.73 (0.64-0.80) | 0.75 (0.63-0.83) | 0.80 (0.68-0.89) | 0.66 (0.52-0.79) |
|  | Agile 3+ | 0.76 (0.67-0.85) | 0.75 (0.65-0.83) | 0.68 (0.59-0.77) | 0.70 (0.57-0.81) | 0.74 (0.60-0.85) |
|  | FIB-4 | 0.69 (0.59-0.79) | 0.78 (0.64-0.88) | 0.58 (0.52-0.65) | 0.47 (0.34-0.60) | 0.85 (0.72-0.93) |
|  | APRI | 0.75 (0.66-0.84) | 0.70 (0.62-0.78) | 0.69 (0.58-0.79) | 0.75 (0.62-0.85) | 0.64 (0.50-0.77) |
| With steatosis | | | | | | |
|  | PRO-C3 (ng/mL) | 0.69 (0.54-0.83) | 0.81 (0.62-0.92) | 0.61 (0.50-0.71) | 0.57 (0.37-0.75) | 0.83 (0.63-0.95) |
|  | GP73 (ng/mL) | 0.80 (0.68-0.93) | 0.83 (0.68-0.91) | 0.76 (0.60-0.87) | 0.80 (0.61-0.92) | 0.79 (0.58-0.93) |
|  | PRO-C3+GP73 | 0.80 (0.68-0.93) | 0.82 (0.66-0.91) | 0.70 (0.56-0.82) | 0.73 (0.54-0.88) | 0.79 (0.58-0.93) |
|  | LSM (kpa) | 0.69 (0.54-0.84) | 0.85 (0.65-0.95) | 0.62 (0.51-0.71) | 0.57 (0.37-0.75) | 0.88 (0.68-0.97) |
|  | FAST | 0.63 (0.48-0.78) | 0.64 (0.54-0.73) | 0.61 (0.42-0.77) | 0.77 (058-0.90) | 0.46 (0.56-0.67) |
|  | Agile 3+ | 0.65 (0.50-0.80) | 0.81 (0.62-0.92) | 0.61 (0.50-0.71) | 0.57 (0.37-0.75) | 0.83 (0.63-0.95) |
|  | FIB-4 | 0.52 (0.36-0.67) | 0.62 (0.49-0.73) | 0.52 (0.38-0.66) | 0.60 (0.41-0.77) | 0.54 (0.33-0.74) |
|  | APRI | 0.65 (0.50-0.80) | 0.75 (0.59-0.86) | 0.60 (0.48-0.71) | 0.60 (0.41-0.77) | 0.75 (0.53-0.90) |
| HBeAg negative | | | | | | |
|  | PRO-C3 (ng/mL) | 0.74 (0.63-0.84) | 0.78 (0.59-0.90) | 0.70 (0.64-0.76) | 0.46 (0.30-0.63) | 0.95 (0.85-0.99) |
|  | GP73 (ng/mL) | 0.69 (0.58-0.80) | 0.55 (0.47-0.59) | 0.77 (0.62-0.87) | 0.79 (0.64-0.91) | 0.55 (0.41-0.68) |
|  | PRO-C3+GP73 | 0.78 (0.68-0.87) | 0.77 (0.61-0.87) | 0.75 (0.67-0.82) | 0.59 (0.42-0.74) | 0.87 (0.76-0.95) |
|  | LSM (kpa) | 0.75 (0.64-0.85) | 0.87 (0.68-0.95) | 0.73 (0.66-0.79) | 0.51 (0.39-0.68) | 0.95 (0.85-0.99) |
|  | FAST | 0.70 (0.59-0.82) | 0.62 (0.51-0.72) | 0.78 (0.67-0.86) | 0.72 (0.55-0.85) | 0.69 (0.55-0.81) |
|  | Agile 3+ | 0.72 (0.61-0.83) | 0.67 (0.53-0.78) | 0.74 (0.65-0.81) | 0.62 (0.45-0.77) | 0.78 (0.65-0.88) |
|  | FIB-4 | 0.59 (0.47-0.71) | 0.71 (0.48-0.86) | 0.65 (0.60-0.70) | 0.31 (0.17-0.48) | 0.91 (0.80-0.97) |
|  | APRI | 0.70 (0.59-0.81) | 0.60 (0.49-0.69) | 0.77 (0.66-0.85) | 0.72 (0.55-0.85) | 0.65 (0.51-0.78) |
| HBeAg positive | | | | | | |
|  | PRO-C3 (ng/mL) | 0.81 (0.70-0.94) | 0.89 (0.80-0.94) | 0.76 (0.57-0.88) | 0.90 (0.79-0.97) | 0.73 (0.80-0.89) |
|  | GP73 (ng/mL) | 0.79 (0.68-0.90) | 0.87 (0.77-0.93) | 0.59 (0.45-0.72) | 0.78 (0.65-0.89) | 0.73 (0.50-0.89) |
|  | PRO-C3+GP73 | 0.84 (0.75-0.94) | 0.97 (0.82-1.00) | 0.51 (0.42-0.60) | 0.61 (0.46-0.74) | 0.95 (0.77-1.00) |
|  | LSM (kpa) | 0.72 (0.60-0.84) | 0.94 (0.80-0.98) | 0.49 (0.40-0.58) | 0.59 (0.44-0.72) | 0.91 (0.71-0.99) |
|  | FAST | 0.67 (0.53-0.81) | 0.79 (0.71-0.85) | 0.59 (0.39-0.77) | 0.86 (0.74-0.94) | 0.45 (0.24-0.68) |
|  | Agile 3+ | 0.80 (0.70-0.90) | 0.94 (0.81-0.98) | 0.53 (0.43-0.62) | 0.65 (0.50-0.78) | 0.91 (0.71-0.99) |
|  | FIB-4 | 0.75 (0.63-0.88) | 0.84 (0.74-0.91) | 0.54 (0.40-0.67) | 0.75 (0.60-0.86) | 0.68 (0.45-0.86) |
|  | APRI | 0.68 (0.54-0.82) | 0.88 (0.74-0.95) | 0.45 (0.36-0.54) | 0.57 (0.42-0.71) | 0.82 (0.60-0.95) |
| HBV DNA < 4 log IU/mL | | | | | | |
|  | PRO-C3 (ng/mL) | 0.70 (0.57-0.83) | 0.53 (0.42-0.63) | 0.81 (0.68-0.89) | 0.74 (0.54-0.89) | 0.62 (0.46-0.76) |
|  | GP73 (ng/mL) | 0.66 (0.53-0.79) | 0.51 (0.42-0.61) | 0.82 (0.68-0.91) | 0.78 (0.58-0.91) | 0.57 (0.42-0.72) |
|  | PRO-C3+GP73 | 0.74 (0.62-0.86) | 0.70 (0.50-0.84) | 0.76 (0.68-0.83) | 0.52 (0.32-0.71) | 0.87 (0.74-0.95) |
|  | LSM (kpa) | 0.68 (0.54-0.82) | 0.80 (0.55-0.93) | 0.75 (0.68-0.81) | 0.44 (0.26-0.65) | 0.94 (0.83-0.98) |
|  | FAST | 0.62 (0.49-0.76) | 0.51 (0.40-0.63) | 0.77 (0.65-0.86) | 0.67 (0.46-0.84) | 0.64 (0.49-0.77) |
|  | Agile 3+ | 0.67 (0.53-0.81) | 0.55 (0.42-0.66) | 0.78(0.67-0.86) | 0.67 (0.46-0.84) | 0.68 (0.53-0.81) |
|  | FIB-4 | 0.52 (0.38-0.66) | 0.89 (0.51-0.98) | 0.61 (0.57-0.64) | 0.15 (0.04-0.34) | 0.98 (0.89-1.00) |
|  | APRI | 0.62 (0.49-0.75) | 0.22 (0.09-0.42) | 0.91 (0.80-0.98) | 0.60 (0.32-0.83) | 0.67 (0.62-0.72) |
| HBV DNA ≥ 4 log IU/mL | | | | | | |
|  | PRO-C3 (ng/mL) | 0.83 (0.74-0.92) | 0.90 (0.78-0.95) | 0.60 (0.49-0.70) | 0.73 (0.60-0.84) | 0.83 (0.65-0.94) |
|  | GP73 (ng/mL) | 0.79 (0.70-0.88) | 0.88 (0.77-0.94) | 0.53 (0.44-0.63) | 0.67 (0.54-0.78) | 0.80 (0.61-0.92) |
|  | PRO-C3+GP73 | 0.86 (0.78 -0.94) | 0.92 (0.82-0.97) | 0.59 (0.49-0.69) | 0.71 (0.59-0.82) | 0.87 (0.69-0.96) |
|  | LSM (kpa) | 0.75 (0.65-0.85) | 0.93 (0.81-0.98) | 0.53 (0.45-0.61) | 0.62 (0.49-0.74) | 0.90 (0.74-0.98) |
|  | FAST | 0.71 (0.60-0.83) | 0.81 (0.72-0.87) | 0.53 (0.41-0.65) | 0.73 (0.60-0.83) | 0.63 (0.44-0.80) |
|  | Agile 3+ | 0.78 (0.69-0.88) | 0.88 (0.78-0.94) | 0.56 (0.45-0.66) | 0.70 (0.57-0.81) | 0.80 (0.61-0.92) |
|  | FIB-4 | 0.72 (0.61-0.83) | 0.82 (0.72-0.88) | 0.51 (0.40-0.62) | 0.70 (0.57-0.81) | 0.67 (0.47-0.83) |
|  | APRI | 0.72 (0.60-0.83) | 0.84 (0.74-0.91) | 0.51 (0.41-0.61) | 0.67 (0.54-0.78) | 0.73 (0.54-0.88) |

Note: Of all patients, 167 have 7 makers (PRO-C3, GP73, PRO-C3+GP73, LSM, FAST, Agile 3+ and FIB-4). ALT normal, ALT ≤35 U/L in the male, ALT ≤25 U/L in the female; ALT elevation, ALT >35 U/L in the male, ALT >25 U/L in the female. Abbreviation: NITs: non-invasive tests; AUC, area under the curve; BMI, body mass index; ALT, alanine aminotransferase; HBeAg, hepatitis B e-antigen; PPV, positive predictive value; NPV, negative predictive value; PRO-C3, N-terminal propeptide of type III collagen; GP73, Golgi protein 73; LSM, liver stiffness measurement; FAST, FibroScan-aspartate aminotransferase; FIB-4, fibrosis-4 index; APRI, aspartate aminotransferase‑to‑platelet ratio index.

**Supplementary Table 6.** Diagnostic accuracy of different NITs for advanced fibrosis under different subgroups by gender, age (years), BMI (kg/m^2^), ALT (U/L), steatosis, HBeAg and HBV DNA (log IU/mL)

| Subgroups | NITs | AUC (95% CI) | PPV (95% CI) | NPV (95% CI) | Sensitivity (95% CI) | Specificity (95% CI) |
| --- | --- | --- | --- | --- | --- | --- |
| Male |  |  |  |  |  |  |
|  | PRO-C3 (ng/mL) | 0.82 (0.73-0.90) | 0.65 (0.52-0.76) | 0.89 (0.81-0.93) | 0.75 (0.57-0.89) | 0.83 (0.72-0.90) |
|  | GP73 (ng/mL) | 0.78 (0.69-0.88) | 0.53 (0.43-0.63) | 0.88 (0.80-0.94) | 0.78 (0.60-0.91) | 0.71 (0.59-0.81) |
|  | PRO-C3+GP73 | 0.86 (0.79-0.93) | 0.68 (0.55-0.78) | 0.93 (0.85-0.97) | 0.84 (0.67-0.95) | 0.83 (0.72-0.90) |
|  | LSM (kpa) | 0.88 (0.81-0.94) | 0.66 (0.53-0.77) | 0.90 (0.82-0.95) | 0.78 (0.60-0.91) | 0.83 (0.72-0.90) |
|  | FAST | 0.80 (0.71-0.88) | 0.47 (0.40-0.84) | 0.95 (0.84-0.99) | 0.94 (0.79-0.99) | 0.55 (0.43-0.66) |
|  | Agile 3+ | 0.82 (0.73-0.91) | 0.63 (0.51-0.73) | 0.90 (0.82-0.94) | 0.78 (0.60-0.91) | 0.80 (0.69-0.88) |
|  | FIB-4 | 0.72 (0.61-0.83) | 0.44 (0.37-0.52) | 0.88 (0.77-0.94) | 0.81 (0.64-0.93) | 0.56 (0.44-0.68) |
|  | APRI | 0.78 (0.69-0.87) | 0.57 (0.44-0.68) | 0.84 (0.77-0.90) | 0.66 (0.47-0.81) | 0.79 (0.68-0.87) |
| Female | | | | | | |
|  | PRO-C3 (ng/mL) | 0.78 (0.65-0.91) | 0.52 (0.40-0.63) | 0.90 (0.77-0.96) | 0.83 (0.59-0.96) | 0.67 (0.51-0.80) |
|  | GP73 (ng/mL) | 0.64 (0.50-0.79) | 0.38 (0.32-0.45) | 0.89 (0.67-0.97) | 0.89 (0.65-0.99) | 0.38 (0.24-0.54) |
|  | PRO-C3+GP73 | 0.78 (0.65-0.91) | 0.52 (0.40-0.63) | 0.90 (0.77-0.96) | 0.83 (0.59-0.96) | 0.67 (0.51-0.80) |
|  | LSM (kpa) | 0.77 (0.64-0.90) | 0.54 (0.40-0.68) | 0.86 (0.74-0.93) | 0.72 (0.47-0.90) | 0.74 (0.58-0.86) |
|  | FAST | 0.71 (0.57-0.85) | 0.48 (0.35-0.62) | 0.83 (0.71-0.91) | 0.67 (0.41-0.87) | 0.69 (0.53-0.82) |
|  | Agile 3+ | 0.73 (0.59-0.86) | 0.53 (0.35-0.69) | 0.81 (0.71-0.88) | 0.56 (0.31-0.79) | 0.79 (0.63-0.90) |
|  | FIB-4 | 0.57 (0.40-0.73) | 0.50 (0.27-0.73) | 0.75 (0.68-0.81) | 0.33 (0.13-0.59) | 0.86 (0.72-0.95) |
|  | APRI | 0.64 (0.48-0.80) | 0.60 (0.39-0.78) | 0.80 (0.71-0.87) | 0.50 (0.26-0.74) | 0.86 (0.72-0.95) |
| Age < 40 years | | | | | | |
|  | PRO-C3 (ng/mL) | 0.84 (0.76-0.92) | 0.63 (0.52-0.74) | 0.92 (0.81-0.97) | 0.86 (0.67-0.96) | 0.76 (0.63-0.86) |
|  | GP73 (ng/mL) | 0.69 (0.58-0.81) | 0.53 (0.41-0.64) | 0.82 (0.72-0.89) | 0.68 (0.48-0.84) | 0.71 (0.57-0.82) |
|  | PRO-C3+GP73 | 0.84 (0.76-0.92) | 0.63 (0.52-0.74) | 0.92 (0.81-0.97) | 0.86 (0.67-0.96) | 0.76 (0.63-0.86) |
|  | LSM (kpa) | 0.82 (0.73-0.92) | 0.56 (0.46-0.65) | 0.91 (0.80-0.96) | 0.86 (0.67-0.96) | 0.67 (0.54-0.79) |
|  | FAST | 0.75 (0.65-0.86) | 0.50 (0.42-0.59) | 0.90 (0.77-0.96) | 0.86 (0.67-0.96) | 0.59 (0.45-0.71) |
|  | Agile 3+ | 0.80 (0.70-0.90) | 0.58 (0.47-0.69) | 0.88 (0.77-0.94) | 0.79 (0.59-0.92) | 0.72 (0.59-0.83) |
|  | FIB-4 | 0.66 (0.54-0.78) | 0.49 (0.37-0.60) | 0.80 (0.70-0.87) | 0.64 (0.44-0.81) | 0.67 (0.54-0.79) |
|  | APRI | 0.70 (0.58-0.82) | 0.45 (0.38-0.53) | 0.45 (0.38-0.53) | 0.86 (0.67-0.96) | 0.50 (0.37-0.63) |
| Age ≥ 40 years | | | | | | |
|  | PRO-C3 (ng/mL) | 0.76 (0.64-0.88) | 0.44 (0.36-0.52) | 0.97 (0.81-1.00) | 0.95 (0.74-1.00) | 0.57 (0.42-0.70) |
|  | GP73 (ng/mL) | 0.78 (0.67-0.89) | 0.42 (0.36-0.49) | 0.97 (0.81-1.00) | 0.95 (0.77-1.00) | 0.51 (0.38-0.64) |
|  | PRO-C3+GP73 | 0.78 (0.67-0.89) | 0.42 (0.36-0.49) | 0.97 (0.81-1.00) | 0.95 (0.77-1.00) | 0.51 (0.38-0.64) |
|  | LSM (kpa) | 0.85 (0.76-0.94) | 0.48 (0.40-0.56) | 0.97 (0.84-1.00) | 0.96 (0.77-1.00) | 0.61 (0.47-0.74) |
|  | FAST | 0.76 (0.64-0.88) | 0.44 (0.35-0.53) | 0.90 (0.78-0.96) | 0.82 (0.60-0.95) | 0.61 (0.47-0.74) |
|  | Agile 3+ | 0.88 (0.79-0.97) | 0.57 (0.46-0.68) | 0.96 (0.85-0.99) | 0.91 (0.71-0.99) | 0.75 (0.62-0.85) |
|  | FIB-4 | 0.78 (0.64-0.91) | 0.57 (0.44-0.69) | 0.90 (0.81-0.95) | 0.77 (0.55-0.92) | 0.78 (0.65-0.88) |
|  | APRI | 0.75 (0.61-0.88) | 0.53 (0.40-0.66) | 0.88 (0.79-0.94) | 0.73 (0.50-0.89) | 0.76 (0.63-0.86) |
| BMI < 23 kg/m^2^ | | | | | | |
|  | PRO-C3 (ng/mL) | 0.81 (0.70-0.92) | 0.44 (0.36-0.52) | 0.97 (0.81-1.00) | 0.95 (0.74-1.00) | 0.57 (0.42-0.70) |
|  | GP73 (ng/mL) | 0.70 (0.57-0.83) | 0.39 (0.31-0.48) | 0.90 (0.76-0.97) | 0.84 (0.60-0.97) | 0.53 (0.39-0.67) |
|  | PRO-C3+GP73 | 0.81 (0.70-0.92) | 0.44 (0.36-0.52) | 0.97 (0.81-1.00) | 0.95 (0.74-1.00) | 0.57 (0.42-0.70) |
|  | LSM (kpa) | 0.88 (0.78-0.97) | 0.59 (0.46-0.71) | 0.95 (0.85-0.99) | 0.89 (0.67-0.99) | 0.77 (0.64-0.88) |
|  | FAST | 0.78 (0.66-0.91) | 0.46 (0.37-0.55) | 0.94 (0.81-0.98) | 0.89 (0.67-0.99) | 0.62 (0.48-0.75) |
|  | Agile 3+ | 0.85 (0.75-0.94) | 0.50 (0.40-0.60) | 0.95 (0.83-0.99) | 0.89 (0.67-0.99) | 0.68 (0.54-0.80) |
|  | FIB-4 | 0.72 (0.59-0.86) | 0.50 (0.34-0.66) | 0.84 (0.75-0.90) | 0.58 (0.34-0.80) | 0.79 (0.66-0.89) |
|  | APRI | 0.77 (0.64-0.90) | 0.65 (0.44-0.81) | 0.86 (0.78-0.91) | 0.58 (0.34-0.80) | 0.89 (0.77-0.96) |
| BMI ≥ 23 kg/m^2^ | | | | | | |
|  | PRO-C3 (ng/mL) | 0.80 (0.70-0.89) | 0.63 (0.51-0.74) | 0.88 (0.79-0.93) | 0.77 (0.59-0.90) | 0.78 (0.66-0.88) |
|  | GP73 (ng/mL) | 0.75 (0.64-0.85) | 0.51 (0.42-0.60) | 0.87 (0.76-0.93) | 0.81 (0.63-0.93) | 0.63 (0.50-0.74) |
|  | PRO-C3+GP73 | 0.80 (0.70-0.89) | 0.63 (0.51-0.74) | 0.88 (0.79-0.93) | 0.77 (0.59-0.90) | 0.78 (0.66-0.88) |
|  | LSM (kpa) | 0.80 (0.70-0.89) | 0.59 (0.47-0.70) | 0.86 (0.77-0.92) | 0.74 (0.55-0.88) | 0.75 (0.63-0.85) |
|  | FAST | 0.73 (0.62-0.84) | 0.51 (0.41-0.61) | 0.83 (0.73-0.90) | 0.71 (0.52-0.86) | 0.67 (0.54-0.78) |
|  | Agile 3+ | 0.76 (0.65-0.86) | 0.56 (0.45-0.67) | 0.85 (0.76-0.91) | 0.74 (0.55-0.88) | 0.72 (0.59-0.82) |
|  | FIB-4 | 0.60 (0.47-0.73) | 0.73 (0.49-0.89) | 0.75 (0.70-0.80) | 0.35 (0.19-0.55) | 0.94 (0.85-0.98) |
|  | APRI | 0.69 (0.57-0.81) | 0.58 (0.44-0.70) | 0.81 (0.72-0.87) | 0.61 (0.42-0.78) | 0.78 (0.66-0.88) |
| ALT normal | | | | | | |
|  | PRO-C3 (ng/mL) | 0.89 (0.81-0.98) | 0.58 (0.41-0.72) | 0.95 (0.84-0.99) | 0.88 (0.62-0.98) | 0.79 (0.65-0.90) |
|  | GP73 (ng/mL) | 0.68 (0.52-0.83) | 0.42 (0.30-0.56) | 0.87 (0.76-0.93) | 0.69 (0.41-0.89) | 0.69 (0.54-0.81) |
|  | PRO-C3+GP73 | 0.89 (0.81-0.98 ) | 0.58 (0.44-0.72) | 0.95 (0.84-0.99) | 0.88 (0.62-0.98) | 0.79 (0.65-0.90) |
|  | LSM (kpa) | 0.78 (0.64-0.92) | 0.48 (0.36-0.61) | 0.92 (0.80-0.97) | 0.81 (0.54-0.96) | 0.71 (0.56-0.83) |
|  | FAST | 0.76 (0.60-0.92) | 0.58 (0.40-0.74) | 0.89 (0.79-0.94) | 0.69 (0.41-0.89) | 0.83 (0.70-0.93) |
|  | Agile 3+ | 0.79 (0.66-0.92) | 0.75 (0.48-0.93) | 0.75 (0.60-0.86) | 0.50 (0.36-0.64) | 0.90 (0.79-0.96) |
|  | FIB-4 | 0.63 (0.44-0.83) | 0.57 (0.35-0.77) | 0.84 (0.76-0.90) | 0.50 (0.25-0.75) | 0.88 (0.75-0.95) |
|  | APRI | 0.71 (0.53-0.89) | 0.88 (0.48-0.98) | 0.84 (0.77-0.89) | 0.44 (0.20-0.70) | 0.98 (0.89-0.99) |
| ALT elevation | | | | | | |
|  | PRO-C3 (ng/mL) | 0.76 (0.66-0.86) | 0.58 (0.47-0.68) | 0.85 (0.76-0.91) | 0.74 (0.56-0.87) | 0.74 (0.62-0.84) |
|  | GP73 (ng/mL) | 0.76 (0.66-0.85) | 0.51 (0.42-0.60) | 0.84 (0.74-0.91) | 0.76 (0.59-0.89) | 0.63 (0.51-0.75) |
|  | PRO-C3+GP73 | 0.76 (0.66-0.86) | 0.58 (0.47-0.68) | 0.85 (0.76-0.91) | 0.74 (0.56-0.87) | 0.74 (0.62-0.84) |
|  | LSM (kpa) | 0.85 (0.78-0.95) | 0.63 (0.52-0.74) | 0.87 (0.78-0.93) | 0.77 (0.59-0.89) | 0.78 (0.67-0.87) |
|  | FAST | 0.77 (0.68-0.87) | 0.60 (0.48-0.71) | 0.84 (0.76-0.90) | 0.71 (0.53-0.85) | 0.77 (0.65-0.86) |
|  | Agile 3+ | 0.82 (0.73-0.90) | 0.65 (0.52-0.76) | 0.85 (0.77-0.91) | 0.71 (0.53-0.85) | 0.81 (0.70-0.90) |
|  | FIB-4 | 0.67 (0.57-0.78) | 0.44 (0.37-0.52) | 0.83 (0.71-0.91) | 0.79 (0.62-0.91) | 0.51 (0.38-0.63) |
|  | APRI | 0.72 (0.62-0.82) | 0.55 (0.45-0.65) | 0.86 (0.76-0.92) | 0.76 (0.59-0.89) | 0.70 (0.57-0.80) |
| Without steatosis | | | | | | |
|  | PRO-C3 (ng/mL) | 0.86 (0.79-0.93) | 0.63 (0.52-0.73) | 0.91 (0.83-0.96) | 0.83 (0.66-0.93) | 0.78 (0.67-0.87) |
|  | GP73 (ng/mL) | 0.73 (0.63-0.83) | 0.51 (0.41-0.61) | 0.84 (0.76-0.90) | 0.71 (0.54-0.85) | 0.69 (0.58-0.79) |
|  | PRO-C3+GP73 | 0.86 (0.79-0.93) | 0.63 (0.52-0.73) | 0.91 (0.83-0.96) | 0.83 (0.66-0.93) | 0.78 (0.67-0.87) |
|  | LSM (kpa) | 0.85 (0.77-0.93) | 0.74 (0.59-0.84) | 0.87 (0.80-0.92) | 0.71 (0.54-0.85) | 0.89 (0.79-0.95) |
|  | FAST | 0.78 (0.69-0.87) | 0.52 (0.44-0.59) | 0.93 (0.83-0.97) | 0.89 (0.73-0.97) | 0.63 (0.51-0.74) |
|  | Agile 3+ | 0.82 (0.74-0.90) | 0.52 (0.44-0.59) | 0.93 (0.83-0.97) | 0.89 (0.73-0.97) | 0.63 (0.51-0.74) |
|  | FIB-4 | 0.68 (0.57-0.78) | 0.59 (0.43-0.74) | 0.78 (0.72-0.83) | 0.46 (0.29-0.63) | 0.86 (0.76-0.93) |
|  | APRI | 0.74 (0.63-0.84) | 0.61 (0.46-0.73) | 0.81 (0.75-0.87) | 0.57 (0.39-0.74) | 0.83 (0.73-0.91) |
| With steatosis | | | | | | |
|  | PRO-C3 (ng/mL) | 0.68 (0.51-0.85) | 0.48 (0.34-0.62) | 0.87 (0.74-0.94) | 0.73 (0.45-0.92) | 0.69 (0.52-0.83) |
|  | GP73 (ng/mL) | 0.74 (0.60-0.88) | 0.44 (0.33-0.56) | 0.89 (0.74-0.96) | 0.80 (0.52-0.96) | 0.62 (0.45-0.77) |
|  | PRO-C3+GP73 | 0.74 (0.60-0.88) | 0.44 (0.33-0.56) | 0.89 (0.74-0.96) | 0.80 (0.52-0.96) | 0.62 (0.45-0.77) |
|  | LSM (kpa) | 0.79 (0.65-0.92) | 0.46 (0.36-0.58) | 0.92 (0.76-0.98) | 0.87 (0.60-0.98) | 0.62 (0.45-0.77) |
|  | FAST | 0.72 (0.55-0.89) | 0.62 (0.38-0.81) | 0.83 (0.74-0.89) | 0.53 (0.27-0.79) | 0.87 (0.73-0.96) |
|  | Agile 3+ | 0.74 (0.58-0.91) | 0.55 (0.39-0.70) | 0.88 (0.76-0.95) | 0.73 (0.45-0.92) | 0.77 (0.61-0.89) |
|  | FIB-4 | 0.59 (0.40-0.79) | 0.39 (0.27-0.51) | 0.82 (0.68-0.91) | 0.67 (0.38-0.88) | 0.59 (0.42-0.74) |
|  | APRI | 0.70 (0.53-0.87) | 0.53 (0.36-0.69) | 0.86 (0.74-0.93) | 0.67 (0.38-0.88) | 0.77 (0.61-0.89) |
| HBeAg negative | | | | | | |
|  | PRO-C3 (ng/mL) | 0.86 (0.76-0.95) | 0.71 (0.53-0.85) | 0.90 (0.84-0.95) | 0.68 (0.45-0.86) | 0.92 (0.83-0.97) |
|  | GP73 (ng/mL) | 0.67 (0.55-0.80) | 0.38 (0.27-0.51) | 0.85 (0.77-0.91) | 0.59 (0.36-0.79) | 0.71 (0.59-0.81) |
|  | PRO-C3+GP73 | 0.86 (0.76-0.95) | 0.71 (0.53-0.85) | 0.90 (0.84-0.95) | 0.68 (0.45-0.86) | 0.92 (0.83-0.97) |
|  | LSM (kpa) | 0.80 (0.68-0.91) | 0.64 (0.46-0.78) | 0.89 (0.82-0.93) | 0.64 (0.41-0.83) | 0.89 (0.79-0.95) |
|  | FAST | 0.75 (0.63-0.87) | 0.41 (0.33-0.50) | 0.92 (0.82-0.97) | 0.82 (0.60-0.95) | 0.64 (0.52-0.75) |
|  | Agile 3+ | 0.79 (0.68-0.90) | 0.39 (0.32-0.46) | 0.95 (0.84-0.99) | 0.91 (0.71-0.99) | 0.56 (0.43-0.67) |
|  | FIB-4 | 0.61 (0.45-0.76) | 0.50 (0.32-0.68) | 0.84 (0.78-0.88) | 0.46 (0.24-0.68) | 0.86 (0.76-0.93) |
|  | APRI | 0.70 (0.57-0.84) | 0.36 (0.28-0.45) | 0.89 (0.79-0.95) | 0.77 (0.55-0.92) | 0.58 (0.46-0.70) |
| HBeAg positive | | | | | | |
|  | PRO-C3 (ng/mL) | 0.69 (0.57-0.82) | 0.55 (0.46-0.63) | 0.86 (0.71-0.94) | 0.86 (0.67-0.96) | 0.56 (0.40-0.70) |
|  | GP73 (ng/mL) | 0.75 (0.64-0.86) | 0.56 (0.47-0.65) | 0.87 (0.72-0.94) | 0.86 (0.67-0.96) | 0.58 (0.42-0.72) |
|  | PRO-C3+GP73 | 0.75 (0.64-0.86) | 0.56 (0.47-0.65) | 0.87 (0.72-0.94) | 0.86 (0.67-0.96) | 0.58 (0.42-0.72) |
|  | LSM (kpa) | 0.84 (0.75-0.93) | 0.60 (0.51-0.68) | 0.96 (0.80-1.00) | 0.96 (0.82-1.00) | 0.60 (0.44-0.74) |
|  | FAST | 0.73 (0.61-0.85) | 0.67 (0.50-0.80) | 0.76 (0.66-0.83) | 0.57 (0.37-0.76) | 0.82 (0.68-0.92) |
|  | Agile 3+ | 0.83 (0.73-0.92) | 0.74 (0.58-0.85) | 0.83 (0.72-0.90) | 0.71 (0.51-0.87) | 0.84 (0.71-0.94) |
|  | FIB-4 | 0.71 (0.59-0.83) | 0.55 (0.45-0.65) | 0.82 (0.68-0.91) | 0.79 (0.59-0.92) | 0.60 (0.44-0.74) |
|  | APRI | 0.70 (0.58-0.83) | 0.61 (0.48-0.72) | 0.80 (0.68-0.88) | 0.71 (0.51-0.87) | 0.71 (0.56-0.84) |
| HBV DNA < 4 log IU/mL | | | | | | |
|  | PRO-C3 (ng/mL) | 0.87 (0.76-0.98) | 0.82 (0.52-0.95) | 0.94 (0.87-0.97) | 0.69 (0.39-0.91) | 0.97 (0.89-1.00) |
|  | GP73 (ng/mL) | 0.66 (0.51-0.81) | 0.35 (0.21-0.52) | 0.89 (0.81-0.94) | 0.54 (0.25-0.81) | 0.79 (0.66-0.88) |
|  | PRO-C3+GP73 | 0.87 (0.76-0.98) | 0.82 (0.52-0.95) | 0.94 (0.87-0.97) | 0.69 (0.39-0.91) | 0.97 (0.89-1.00) |
|  | LSM (kpa) | 0.77 (0.59-0.94) | 0.73 (0.45-0.90) | 0.92 (0.85-0.96) | 0.62 (0.32-0.86) | 0.95 (0.86-0.99) |
|  | FAST | 0.71 (0.55-0.86) | 0.31 (0.21-0.43) | 0.91 (0.82-0.96) | 0.69 (0.39-0.91) | 0.67 (0.54-0.79) |
|  | Agile 3+ | 0.79 (0.62-0.95) | 0.53 (0.34-0.72) | 0.92 (0.84-0.96) | 0.62 (0.32-0.86) | 0.89 (0.78-0.95) |
|  | FIB-4 | 0.60 (0.39-0.81) | 0.80 (0.33-0.97) | 0.87 (0.82-0.91) | 0.30 (0.09-0.61) | 0.98 (0.91-1.00) |
|  | APRI | 0.63 (0.45-0.81) | 0.25 (0.16-0.36) | 0.88 (0.78-0.94) | 0.62 (0.32-0.86) | 0.61 (0.47-0.73) |
| HBV DNA ≥ 4 log IU/mL | | | | | | |
|  | PRO-C3 (ng/mL) | 0.72 (0.61-0.82) | 0.58 (0.49-0.68) | 0.80 (0.69-0.88) | 0.76 (0.59-0.88) | 0.64 (0.50-0.77) |
|  | GP73 (ng/mL) | 0.74 (0.63-0.84) | 0.56 (0.47-0.65) | 0.79 (0.67-0.87) | 0.76 (0.59-0.88) | 0.61 (0.47-0.74) |
|  | PRO-C3+GP73 | 0.74 (0.63-0.84) | 0.56 (0.47-0.65) | 0.79 (0.67-0.87) | 0.76 (0.59-0.88) | 0.61 (0.47-0.74) |
|  | LSM (kpa) | 0.82 (0.74-0.90) | 0.62 (0.54-0.70) | 0.97 (0.83-1.00) | 0.97 (0.86-1.00) | 0.61 (0.47-0.74) |
|  | FAST | 0.73 (0.63-0.84) | 0.55 (0.48-0.62) | 0.90 (0.75-0.97) | 0.92 (0.78-0.98) | 0.50 (0.36-0.64) |
|  | Agile 3+ | 0.80 (0.71-0.89) | 0.69 (0.57-0.79) | 0.82 (0.72-0.88) | 0.73 (0.56-0.86) | 0.79 (0.66-0.88) |
|  | FIB-4 | 0.67 (0.56-0.78) | 0.52 (0.44-0.60) | 0.78 (0.65-0.88) | 0.78 (0.62-0.90) | 0.52 (0.38-0.65) |
|  | APRI | 0.71 (0.60-0.82) | 0.62 (0.51-0.72) | 0.78 (0.68-0.86) | 0.70 (0.53-0.84) | 0.71 (0.58-0.83) |

Note: Of all patients, 167 have 7 makers (PRO-C3, GP73, PRO-C3+GP73, LSM, FAST, Agile 3+ and FIB-4). ALT normal, ALT ≤35 U/L in the male, ALT ≤25 U/L in the female; ALT elevation, ALT >35 U/L in the male, ALT >25 U/L in the female. Abbreviation: AUC, area under the curve; BMI, body mass index; ALT, alanine aminotransferase; HBeAg, hepatitis B e-antigen; PPV, positive predictive value; NPV, negative predictive value; PRO-C3, N-terminal propeptide of type III collagen; GP73, Golgi protein 73; LSM, liver stiffness measurement; FAST, FibroScan-aspartate aminotransferase; FIB-4, fibrosis-4 index; APRI, aspartate aminotransferase‑to‑platelet ratio index.

**Supplementary Table 7.** Delong test for the differences between AUCs of the different NITs under different subgroups by gender, age (years), BMI (kg/m^2^), ALT (U/L), steatosis, HBeAg and HBV DNA (log IU/mL)

|  | *P*-value compared to  PRO-C3 | *P*-value compared to  GP73 | *P*-value  compared to  PRO-C3 + GP73 | *P*-value compared to  LSM | *P*-value compared to  FAST | *P*-value compared to  Agile 3+ | *P*-value compared to  FIB-4 | *P*-value compared to  APRI |
| --- | --- | --- | --- | --- | --- | --- | --- | --- |
| Male |  |  |  |  |  |  |  |  |
| PRO-C3 | 1 | 0.12^b^ | 1.00^b^ | 0.94^b^ | 0.46^b^ | 0.63^b^ | 0.20^b^ | 0.54^b^ |
| GP73 | 0.58^a^ | 1 | 0.12^b^ | 0.11^b^ | 0.43^b^ | 0.34^b^ | 0.39^b^ | 0.98^b^ |
| PRO-C3 + GP73 | 0.26^a^ | 0.10^a^ | 1 | 0.94^b^ | 0.46^b^ | 0.63^b^ | 0.04^b^ | 0.13^b^ |
| LSM | 0.66^a^ | 0.89^a^ | 0.30^a^ | 1 | 0.27^b^ | 0.46^b^ | <0.01^b^ | 0.03^b^ |
| FAST | 0.18^a^ | 0.55^a^ | 0.04^a^ | 0.29^a^ | 1 | 0.82^b^ | 0.19^b^ | 0.49^b^ |
| Agile 3+ | 0.34^a^ | 0.68^a^ | 0.12^a^ | 0.26^a^ | 0.86^a^ | 1 | 0.01^b^ | 0.95^b^ |
| FIB-4 | 0.02^a^ | 0.07^a^ | <0.01^a^ | 0.01^a^ | 0.13^a^ | 0.02^a^ | 1 | 0.20^b^ |
| APRI | 0.11^a^ | 0.36^a^ | 0.02^a^ | 0.18^a^ | 0.48^a^ | 0.59^a^ | 0.18^a^ | 1 |
| Female |  |  |  |  |  |  |  |  |
| PRO-C3 | 1 | / | 1.00^b^ | 0.94^b^ | 0.46^b^ | 0.63^b^ | / | / |
| GP73 | 0.23^a^ | 1 | / | / | / | / | / | / |
| PRO-C3 + GP73 | 1.00^a^ | 0.23^a^ | 1 | 0.94^b^ | 0.46^b^ | 0.63^b^ | / | / |
| LSM | 0.26^a^ | 0.97^a^ | 0.26^a^ | 1 | 0.27^b^ | 0.46^b^ | / | / |
| FAST | 0.40^a^ | 0.67^a^ | 0.40^a^ | 0.58^a^ | 1 | 0.82^b^ | / | / |
| Agile 3+ | 0.13^a^ | 0.61^a^ | 0.13^a^ | 0.53^a^ | 0.28^a^ | 1 | / | / |
| FIB-4 | / | / | / | / | / | / | 1 | / |
| APRI | 0.46^a^ | 0.66^a^ | 0.46^a^ | 0.64^a^ | 0.94^a^ | 0.28^a^ | / | 1 |
| Age < 40 years |  |  |  |  |  |  |  |  |
| PRO-C3 | 1 | 0.02^b^ | 1.00^b^ | 0.77^b^ | 0.17^b^ | 0.50^b^ | 0.01^b^ | 0.03^b^ |
| GP73 | 0.57^a^ | 1 | 0.02^b^ | 0.05^b^ | 0.36^b^ | 0.14^b^ | 0.67^b^ | 0.97^b^ |
| PRO-C3 + GP73 | 0.11^a^ | 0.07^a^ | 1 | 0.77^b^ | 0.17^b^ | 0.50^b^ | 0.01^b^ | 0.03^b^ |
| LSM | 0.11^a^ | 0.33^a^ | 0.01^a^ | 1 | 0.12^b^ | 0.56^b^ | 0.01^b^ | 0.03^b^ |
| FAST | 0.06^a^ | 0.28^a^ | <0.01^a^ | 0.92^a^ | 1 | 0.51^b^ | 0.11^b^ | 0.06^b^ |
| Agile 3+ | 0.30^a^ | 0.67^a^ | 0.06^a^ | 0.36^a^ | 0.49^a^ | 1 | 0.01^b^ | 0.15^b^ |
| FIB-4 | 0.06^a^ | 0.26^a^ | <0.01^a^ | 0.83^a^ | 0.87^a^ | 0.28^a^ | 1 | 0.43^b^ |
| APRI | 0.06^a^ | 0.24^a^ | <0.01^a^ | 0.79^a^ | 0.74^a^ | 0.40^a^ | 0.96^a^ | 1 |
| Age ≥ 40 years |  |  |  |  |  |  |  |  |
| PRO-C3 | 1 | 0.74^b^ | 0.74^b^ | 0.21^b^ | 0.99^b^ | 0.10^b^ | 0.83^b^ | 0.92^b^ |
| GP73 | 0.21^a^ | 1 | 1.00^b^ | 0.29^b^ | 0.72^b^ | 0.15^b^ | 0.94^b^ | 0.64^b^ |
| PRO-C3 + GP73 | 1.00^a^ | 0.31^a^ | 1 | 0.29^b^ | 0.72^b^ | 0.15^b^ | 0.94^b^ | 0.62^b^ |
| LSM | 0.78^a^ | 0.48^a^ | 0.78^a^ | 1 | 0.04^b^ | 0.47^b^ | 0.27^b^ | 0.11^b^ |
| FAST | 0.48^a^ | 0.73^a^ | 0.48^a^ | 0.58^a^ | 1 | 0.01^b^ | 0.77^b^ | 0.83^b^ |
| Agile 3+ | 0.65^a^ | 0.59^a^ | 0.65^a^ | 0.77^a^ | 0.82^a^ | 1 | 0.02^b^ | 0.01^b^ |
| FIB-4 | 0.12^a^ | 0.56^a^ | 0.12^a^ | 0.15^a^ | 0.30^a^ | 0.04^a^ | 1 | 0.42^b^ |
| APRI | 0.42^a^ | 0.86^a^ | 0.42^a^ | 0.55^a^ | 0.80^a^ | 0.66^a^ | 0.20^a^ | 1 |
| BMI < 23 kg/m^2^ |  |  |  |  |  |  |  |  |
| PRO-C3 | 1 | 0.18^b^ | 1.00^b^ | 0.42^b^ | 0.76^b^ | 0.67^b^ | 0.38^b^ | 0.66^b^ |
| GP73 | 0.02^a^ | 1 | 0.18^b^ | 0.03^b^ | 0.33^b^ | 0.07^b^ | 0.80^b^ | 0.44^b^ |
| PRO-C3 + GP73 | 1.00^a^ | 0.02^a^ | 1 | 0.42^b^ | 0.76^b^ | 0.67^b^ | 0.38^b^ | 0.66^b^ |
| LSM | 0.08^a^ | 0.78^a^ | 0.08^a^ | 1 | 0.06^b^ | 0.54^b^ | 0.04^b^ | 0.13^b^ |
| FAST | 0.11^a^ | 0.50^a^ | 0.11^a^ | 0.65^a^ | 1 | 0.37^b^ | 0.40^b^ | 0.76^b^ |
| Agile 3+ | 0.07^a^ | 0.85^a^ | 0.07^a^ | 0.89^a^ | 0.62^a^ | 1 | 0.02^b^ | 0.29^b^ |
| FIB-4 | 0.06^a^ | 0.86^a^ | 0.06^a^ | 0.90^a^ | 0.54^a^ | 0.97^a^ | 1 | 0.40^b^ |
| APRI | 0.16^a^ | 0.46^a^ | 0.16^a^ | 0.62^a^ | 0.81^a^ | 0.54^a^ | 0.35^a^ | 1 |
| BMI ≥ 23 kg/m^2^ |  |  |  |  |  |  |  |  |
| PRO-C3 | 1 | 0.42^b^ | 1.00^b^ | 0.97^b^ | 0.24^b^ | 0.56^b^ | / | 0.09^b^ |
| GP73 | 0.67^a^ | 1 | 0.42^b^ | 0.42^b^ | 0.77^b^ | 0.90^b^ | / | 0.33^b^ |
| PRO-C3 + GP73 | 0.16^a^ | 0.39^a^ | 1 | 0.97^b^ | 0.24^b^ | 0.56^b^ | / | 0.09^b^ |
| LSM | 0.73^a^ | 0.45^a^ | 0.20^a^ | 1 | 0.12^b^ | 0.31^b^ | / | 0.047^b^ |
| FAST | 0.16^a^ | 0.12^a^ | 0.02^a^ | 0.19^a^ | 1 | 0.68^b^ | / | 0.21^b^ |
| Agile 3+ | 0.54^a^ | 0.26^a^ | 0.11^a^ | 0.61^a^ | 0.57^a^ | 1 | / | 0.31^b^ |
| FIB-4 | / | / | / | / | / | / | 1 | / |
| APRI | 0.19^a^ | 0.10^a^ | 0.02^a^ | 0.27^a^ | 1.00^a^ | 0.56^a^ | / | 1 |
| ALT normal |  |  |  |  |  |  |  |  |
| PRO-C3 | 1 | 0.02^b^ | 1.00^b^ | 0.21^b^ | 0.19^b^ | 0.22^b^ | / | 0.09^b^ |
| GP73 | 0.04^a^ | 1 | 0.02^b^ | 0.26^b^ | 0.41^b^ | 0.20^b^ | / | 0.78^b^ |
| PRO-C3 + GP73 | 1.00^a^ | 0.04^a^ | 1 | 0.19^b^ | 0.22^b^ | 0.71^b^ | / | 0.09^b^ |
| LSM | 0.20^a^ | 0.61^a^ | 0.20^a^ | 1 | 0.85^b^ | 0.59^b^ | / | 0.50^b^ |
| FAST | 0.19^a^ | 0.54^a^ | 0.19^a^ | 0.93^a^ | 1 | 0.42^b^ | / | 0.55^b^ |
| Agile 3+ | 0.53^a^ | 0.17^a^ | 0.53^a^ | 0.24^a^ | 0.41^a^ | 1 | / | 0.24^b^ |
| FIB-4 | 0.06^a^ | 0.86^a^ | 0.06^a^ | 0.53^a^ | 0.43^a^ | 0.04^a^ | 1 | / |
| APRI | 0.28^a^ | 0.42^a^ | 0.28^a^ | 0.84^a^ | 0.85^a^ | 0.53^a^ | 0.14^a^ | 1 |
| ALT elevation |  |  |  |  |  |  |  |  |
| PRO-C3 | 1 | 0.92^b^ | 1.00^b^ | 0.14^b^ | 0.85^b^ | 0.42^b^ | 0.26^b^ | 0.48^b^ |
| GP73 | 0.96^a^ | 1 | 0.92^b^ | 0.09^b^ | 0.76^b^ | 0.31^b^ | 0.19^b^ | 0.51^b^ |
| PRO-C3 + GP73 | 0.09^a^ | 0.13^a^ | 1 | 0.14^b^ | 0.85^b^ | 0.42^b^ | 0.22^b^ | 0.48^b^ |
| LSM | 0.38^a^ | 0.38^a^ | 0.06^a^ | 1 | 0.06^b^ | 0.24^b^ | <0.001^b^ | 0.01^b^ |
| FAST | 0.09^a^ | 0.11^a^ | <0.01^a^ | 0.32^a^ | 1 | 0.40^b^ | 0.03^b^ | 0.04^b^ |
| Agile 3+ | 0.32^a^ | 0.29^a^ | 0.04^a^ | 0.76^a^ | 0.55^a^ | 1 | <0.001^b^ | 0.07^b^ |
| FIB-4 | 0.03^a^ | 0.02^a^ | <0.001^a^ | 0.07^a^ | 0.21^a^ | 0.02^a^ | 1 | 0.23^b^ |
| APRI | 0.07^a^ | 0.08^a^ | <0.01^a^ | 0.26^a^ | 0.57^a^ | 0.36^a^ | 0.24^a^ | 1 |
| Without steatosis |  |  |  |  |  |  |  |  |
| PRO-C3 | 1 | 0.02^b^ | 1.00^b^ | 0.89^b^ | 0.13^b^ | 0.48^b^ | <0.01^b^ | 0.03^b^ |
| GP73 | 0.01^a^ | 1 | 0.02^b^ | 0.02^b^ | 0.32^b^ | 0.08^b^ | 0.41^b^ | 0.89^b^ |
| PRO-C3 + GP73 | 0.42^a^ | <0.01^a^ | 1 | 0.89^b^ | 0.13^b^ | 0.48^b^ | <0.01^b^ | 0.03^b^ |
| LSM | 0.06^a^ | 0.48^a^ | 0.03^a^ | 1 | 0.04^b^ | 0.37^b^ | <0.01^b^ | 0.02^b^ |
| FAST | 0.05^a^ | 0.44^a^ | 0.03^a^ | 0.91^a^ | 1 | 0.41^b^ | 0.049^b^ | 0.16^b^ |
| Agile 3+ | 0.06^a^ | 0.51^a^ | 0.03^a^ | 0.94^a^ | 0.89^a^ | 1 | <0.001^b^ | 0.10^b^ |
| FIB-4 | <0.01^a^ | 0.58^a^ | <0.001^a^ | 0.19^a^ | 0.15^a^ | 0.06^a^ | 1 | 0.16^b^ |
| APRI | 0.03^a^ | 0.70^a^ | 0.01^a^ | 0.75^a^ | 0.54^a^ | 0.80^a^ | 0.17^a^ | 1 |
| With steatosis |  |  |  |  |  |  |  |  |
| PRO-C3 | 1 | 0.57^b^ | 0.57^b^ | 0.35^b^ | 0.73^b^ | 0.65^b^ | / | 0.85^b^ |
| GP73 | 0.22^a^ | 1 | 1.00^b^ | 0.59^b^ | 0.84^b^ | 0.95^b^ | / | 0.73^b^ |
| PRO-C3 + GP73 | 0.22^a^ | 1.00^a^ | 1 | 0.59^b^ | 0.84^b^ | 0.95^b^ | / | 0.73^b^ |
| LSM | 0.99^a^ | 0.29^a^ | 0.29^a^ | 1 | 0.30^b^ | 0.55^b^ | / | 0.29^b^ |
| FAST | / | / | / | / | 1 | 0.81^b^ | / | 0.69^b^ |
| Agile 3+ | / | / | / | / | / | 1 | / | 0.67^b^ |
| FIB-4 | / | / | / | / | / | / | 1 | / |
| APRI | / | / | / | / | / | / | / | 1 |
| HBeAg negative |  |  |  |  |  |  |  |  |
| PRO-C3 | 1 | 0.03^b^ | 1.00^b^ | 0.45^b^ | 0.15^b^ | 0.39^b^ | / | 0.06^b^ |
| GP73 | 0.56^a^ | 1 | 0.03^b^ | 0.08^b^ | 0.26^b^ | 0.08^b^ | / | 0.68^b^ |
| PRO-C3 + GP73 | 0.22^a^ | 0.17^a^ | 1 | 0.45^b^ | 0.15^b^ | 0.39^b^ | / | 0.06^b^ |
| LSM | 0.90^a^ | 0.44^a^ | 0.67^a^ | 1 | 0.21^b^ | 0.85^b^ | / | 0.20^b^ |
| FAST | 0.64^a^ | 0.86^a^ | 0.30^a^ | 0.37^a^ | 1 | 0.52^b^ | / | 0.43^b^ |
| Agile 3+ | 0.79^a^ | 0.72^a^ | 0.40^a^ | 0.47^a^ | 0.84^a^ | 1 | / | 0.21^b^ |
| FIB-4 | / | / | / | / | / | / | 1 | / |
| APRI | 0.60^a^ | 0.93^a^ | 0.26^a^ | 0.44^a^ | 0.89^a^ | 0.76^a^ | / | 1 |
| HBeAg positive |  |  |  |  |  |  |  |  |
| PRO-C3 | 1 | 0.38^b^ | 0.39^b^ | 0.049^b^ | 0.64^b^ | 0.08^b^ | 0.85^b^ | 0.90^b^ |
| GP73 | 0.79^a^ | 1 | 1.00^b^ | 0.19^b^ | 0.81^b^ | 0.25^b^ | 0.58^b^ | 0.55^b^ |
| PRO-C3 + GP73 | 0.43^a^ | 0.31^a^ | 1 | 0.19^b^ | 0.81^b^ | 0.25^b^ | 0.58^b^ | 0.55^b^ |
| LSM | 0.22^a^ | 0.41^a^ | 0.10^a^ | 1 | 0.04^b^ | 0.76^b^ | 0.2^b^ | 0.02^b^ |
| FAST | 0.05^a^ | 0.15^a^ | 0.01^a^ | 0.41^a^ | 1 | 0.14^b^ | 0.67^b^ | 0.31^b^ |
| Agile 3+ | 0.84^a^ | 0.91^a^ | 0.48^a^ | 0.12^a^ | 0.06^a^ | 1 | 0.01^b^ | 0.06^b^ |
| FIB-4 | 0.41^a^ | 0.59^a^ | 0.14^a^ | 0.61^a^ | 0.14^a^ | 0.34^a^ | 1 | 0.91^b^ |
| APRI | 0.07^a^ | 0.16^a^ | 0.01^a^ | 0.52^a^ | 0.90^a^ | 0.07^a^ | 0.049^a^ | 1 |
| HBV DNA < 4 Log IU/mL | | | | | | | |  |
| PRO-C3 | 1 | 0.04^b^ | 1.00^b^ | 0.38^b^ | 0.13^b^ | 0.45^b^ | / | / |
| GP73 | 0.67^a^ | 1 | 0.04^b^ | 0.29^b^ | 0.57^b^ | 0.22^b^ | / | / |
| PRO-C3 + GP73 | 0.33^a^ | 0.32^a^ | 1 | 0.38^b^ | 0.13^b^ | 0.45^b^ | / | / |
| LSM | 0.81^a^ | 0.84^a^ | 0.51^a^ | 1 | 0.38^b^ | 0.77^b^ | / | / |
| FAST | / | / | / | / | 1 | 0.42^b^ | / | / |
| Agile 3+ | 0.72^a^ | 0.95^a^ | 0.42^a^ | 0.83^a^ | / | 1 | / | / |
| FIB-4 | / | / | / | / | / | / | 1 | / |
| APRI | / | / | / | / | / | / | / | 1 |
| HBV DNA ≥ 4 Log IU/mL | | | | | | | |  |
| PRO-C3 | 1 | 0.74^b^ | 0.74^b^ | 0.08^b^ | 0.78^b^ | 0.21^b^ | 0.55^b^ | 0.92^b^ |
| GP73 | 0.55^a^ | 1 | 1.00^b^ | 0.16^b^ | 0.98^b^ | 0.32^b^ | 0.35^b^ | 0.69^b^ |
| PRO-C3 + GP73 | 0.30^a^ | 0.12^a^ | 1 | 0.16^b^ | 0.98^b^ | 0.32^b^ | 0.35^b^ | 0.69^b^ |
| LSM | 0.19^a^ | 0.59^a^ | 0.08^a^ | 1 | 0.04^b^ | 0.53^b^ | <0.01^b^ | 0.02^b^ |
| FAST | 0.04^a^ | 0.27^a^ | 0.01^a^ | 0.42^a^ | 1 | 0.25^b^ | 0.22^b^ | 0.35^b^ |
| Agile 3+ | 0.46^a^ | 0.90^a^ | 0.21^a^ | 0.48^a^ | 0.27^a^ | 1 | <0.001^b^ | 0.11^b^ |
| FIB-4 | 0.11^a^ | 0.33^a^ | 0.02^a^ | 0.64^a^ | 0.87^a^ | 0.16^a^ | 1 | 0.36^b^ |
| APRI | 0.07^a^ | 0.31^a^ | 0.01^a^ | 0.59^a^ | 0.87^a^ | 0.34^a^ | 0.93^a^ | 1 |

Note: Of all patients, 167 have 7 makers (PRO-C3, GP73, PRO-C3+GP73, LSM, FAST, Agile 3+，and FIB-4). ALT normal, ALT ≤35 U/L in the male, ALT ≤25 U/L in the female; ALT elevation, ALT >35 U/L in the male, ALT >25 U/L in the female. “/” indicates no comparison was performed due to no statistical difference for AUC in one subgroup. Abbreviation: AUC, area under the curve; NITs: non-invasive tests; BMI, body mass index; ALT, alanine aminotransferase; HBeAg, hepatitis B e-antigen; PRO-C3, N-terminal propeptide of type III collagen; GP73, Golgi protein 73; LSM, liver stiffness measurement; FAST, FibroScan-aspartate aminotransferase; FIB-4, fibrosis-4 index; APRI, aspartate aminotransferase‑to‑platelet ratio index.
